# The health of adolescents in detention: a global scoping review

**DOI:** 10.1016/S2468-2667(19)30217-8

**Published:** 2020-01-16

**Authors:** Rohan Borschmann, Emilia Janca, Annie Carter, Melissa Willoughby, Nathan Hughes, Kathryn Snow, Emily Stockings, Nicole T M Hill, Jane Hocking, Alexander Love, George C Patton, Susan M Sawyer, Seena Fazel, Cheneal Puljević, Jo Robinson, Stuart A Kinner

**Affiliations:** Justice Health Unit, Centre for Health Equity, Melbourne School of Population and Global Health; Melbourne School of Psychological Sciences; The University of Melbourne, Melbourne, VIC, Australia; Centre for Adolescent Health; Murdoch Children’s Research Institute, Melbourne, VIC, Australia; Health Service and Population Research Department, Institute of Psychiatry, Psychology, and Neuroscience, King’s College London, London, UK; Justice Health Unit, Centre for Health Equity, Melbourne School of Population and Global Health; The University of Melbourne, Melbourne, VIC, Australia; Centre for Adolescent Health; Murdoch Children’s Research Institute, Melbourne, VIC, Australia; The University of Melbourne, Melbourne, VIC, Australia; Centre for Adolescent Health; Murdoch Children’s Research Institute, Melbourne, VIC, Australia; Department of Sociological Studies, University of Sheffield, Sheffield, UK; Justice Health Unit, Centre for Health Equity, Melbourne School of Population and Global Health; Centre for International Child Health, Department of Paediatrics; National Drug and Alcohol Research Centre, University of New South Wales Sydney, Sydney, NSW, Australia; Orygen Youth Health, Melbourne, VIC, Australia; Sexual Health Unit, Centre for Epidemiology and Biostatistics, Melbourne School of Population and Global Health; Justice Health Unit, Centre for Health Equity, Melbourne School of Population and Global Health; The University of Melbourne, Melbourne, VIC, Australia; Centre for Adolescent Health; Murdoch Children’s Research Institute, Melbourne, VIC, Australia; Department of Paediatrics; The University of Melbourne, Melbourne, VIC, Australia; Centre for Adolescent Health; Murdoch Children’s Research Institute, Melbourne, VIC, Australia; Royal Children’s Hospital, Melbourne, VIC, Australia; Department of Psychiatry, University of Oxford, Warneford Hospital, Oxford, UK; Centre for Health Services Research, Faculty of Medicine; Orygen Youth Health, Melbourne, VIC, Australia; Justice Health Unit, Centre for Health Equity, Melbourne School of Population and Global Health; The University of Melbourne, Melbourne, VIC, Australia; Centre for Adolescent Health; Murdoch Children’s Research Institute, Melbourne, VIC, Australia; Mater Research Institute-UQ; University of Queensland, Brisbane, QLD, Australia; Griffith Criminology Institute, Griffith University, Brisbane, QLD, Australia; School of Public Health and Preventive Medicine, Monash University, Melbourne, VIC, Australia

## Abstract

Adolescents detained within the criminal justice system are affected by complex health problems, health-risk behaviours, and high rates of premature death. We did a global synthesis of the evidence regarding the health of this population. We searched Embase, PsycINFO, Education Resources Information Center, PubMed, Web of Science, CINCH, Global Health, the Cochrane Database of Systematic Reviews, the Campbell Library, the National Criminal Justice Reference System Abstract Database, and Google Scholar for peer-reviewed journal articles, including reviews, that reported the prevalence of at least one health outcome (physical, mental, sexual, infectious, and neurocognitive) in adolescents (aged <20 years) in detention, and were published between Jan 1, 1980, and June 30, 2018. The reference lists of published review articles were scrutinised for additional relevant publications. Two reviewers independently screened titles and abstracts, and three reviewed full texts of relevant articles. The protocol for this Review was registered with PROSPERO (CRD42016041392). 245 articles (204 primary research articles and 41 reviews) were included, with most primary research (183 [90%]) done in high-income countries. A high lifetime prevalence of health problems, risks, and conditions was reported in detained adolescents, including mental disorders (0–95%), substance use disorders (22–96%), self-harm (12–65%), neurodevelopmental disabilities (2–47%), infectious diseases (0–34%), and sexual and reproductive conditions (pregnant by age 19 years 20–37%; abnormal cervical screening test result 16%). Various physical and mental health problems and health-risk behaviours are more common among adolescents in detention than among their peers who have not been detained. As the social and structural drivers of poor health overlap somewhat with factors associated with exposure to the criminal justice system, strategies to address these factors could help to reduce both rates of adolescent detention and adolescent health inequalities. Improving the detection of mental and physical disorders, providing appropriate interventions during detention, and optimising transitional health care after release from detention could improve the health outcomes of these vulnerable young people.

## Introduction

The life trajectories of many adolescents detained within the criminal justice system are characterised by entrenched disadvantage, instability, abuse, neglect, poor education, and poverty.^1–3^ These social and structural drivers of detention overlap to a large degree with the determinants of early disease morbidity and mortality. Growing evidence suggests that adolescents who have been in detention die at a rate that is five to 41 times higher than that of their age-matched and sex-matched peers, most often from drug overdose, suicide, injury, or violence.^4–7^ Many detained adolescents also have complex, co-occurring health conditions, such as mental disorder^8,9^ (including self-harm,^10^ suicidal behaviour,^11^ and substance dependence),^12^ cognitive dysfunction and learning difficulties,^13^ non-communicable diseases (eg, asthma),^14^ and sexually transmitted infections (STIs) and blood-borne viral infections.^15^ Many individuals under-utilise primary and preventive care in the community,^16^ such that detention often represents the first meaningful opportunity to identify their physical and mental health needs and to initiate appropriate health care.

Effective care planning and coordination requires an understanding of the prevalence and co-occurrence of health problems, but global evidence regarding the health of detained adolescents has never been fully synthesised. Previous reviews have focused on one health condition (eg, mental disorder)^8,9^ or synthesised evidence across health domains for one country.^1^ The most comprehensive review of detained adolescents^1^ focused solely on US studies and was published more than a decade ago. In addition to documenting markedly elevated rates of morbidity and mortality among this population, the authors identified a high prevalence of health-compromising behaviours, and a distinct lack of familial and community supports to facilitate reintegration into the community after release from detention.^1^ There remains a pressing need to synthesise the findings of studies done in other settings.^17^ In this global Scoping Review, we aimed to synthesise the evidence regarding the health of adolescents detained within the criminal justice system in any country. This included both youth and adult criminal justice systems, provided that the age criterion was met.

## Methods

### Overview

We conducted a systematic search to identify literature on the health of detained adolescents. Our Scoping Review was done in accordance with the Preferred Reporting Items for Systematic Reviews and Meta-Analyses Extension for Scoping Reviews guidelines.^18^ The protocol was registered with the PROSPERO (number CRD42016041392) before the review was done.

### Search strategy and selection criteria

We searched 11 electronic databases: Embase, PsycINFO, Education Resources Information Center, PubMed, Web of Science, CINCH, Global Health, the Cochrane Database of Systematic Reviews, the Campbell Library, the National Criminal Justice Reference System Abstract Database, and Google Scholar. We used variants and combinations of search terms relating to custody or detention under the criminal justice system and physical, mental, sexual, infectious, and neurocognitive health conditions (appendix pp 1–4). All databases were searched on March 1, 2017, for entries from Jan 1, 1980, to Feb 28, 2017, and the search was updated on July 1, 2018, by a rapid review for entries to June 30, 2018. We scrutinised the reference lists of published review articles to locate additional relevant publications not identified during the database searches. We also corresponded with experts in the field to identify additional publications.

Publication format was limited to peer-reviewed journal articles (as a filter for quality resulting from the peer-review process), including all types of review publications (narrative, systematic, and meta-analysis). The rationale for including previous reviews was that scoping reviews are designed to identify key themes and trends in the literature,^19^ as opposed to extracting data for meta-analysis, and previous reviews are valuable sources of such themes. We included publications from any country and in any language. Publications were deemed eligible for inclusion if participants had been detained within the criminal justice system. Because not all countries have separate youth and adult criminal justice systems and the age cutoff between youth and adult detention varies between countries, publications relating to adolescents (aged <20 years, as defined by the UN^20^ and used in a previous large review)^9^ incarcerated in adult correctional institutions were included, if findings were appropriately disaggregated by age. Only publications in which all participants were younger than 20 years of age at the time they were detained, and which reported the prevalence of at least one health outcome, were eligible for inclusion. Studies were excluded if they reported on health outcomes in selected samples only (eg, adolescents detained in psychiatric hospitals or those referred to health care). We also excluded studies that reported knowledge of health-risk behaviours or intention to engage in health-protective behaviours (eg, condom use) but did not report on an actual health outcome.

### Publication selection

Search results were imported into EndNote X8 reference management software and duplicates were deleted. Title and abstract screening was done independently by two researchers (including EJ). Full-text reviews of the remaining publications were then done independently by three researchers (including EJ and CP) and reference lists of potentially relevant publications were manually searched. Uncertainty regarding whether publications met the inclusion criteria was resolved through discussion among the three researchers. In instances when the full text of potentially relevant publications could not be located, two attempts were made to contact the author(s) via email to request a copy.

### Quality assessment

The Joanna Briggs Institute Critical Appraisal Checklist for Prevalence Studies^21^ was used to assess the methodological quality of all primary research publications by evaluating the extent to which they addressed the possibility of bias in nine areas of study design, conduct, and analysis. Each of the nine domains received a score from 0 (poor quality) to 2 (high quality), and a total quality score was calculated by summing the individual domain scores. Total scores ranged from 0 to 18, with higher scores indicating higher quality. Studies with a total score of less than 13 were excluded. Four researchers (EJ, CP, MW, AL) independently assessed each included publication and any uncertainty regarding the quality of publications was resolved through discussion among them.

### Role of the funding source

The funder of the study had no role in study design, data collection and analysis, decision to publish, or preparation of the manuscript.

## Results

The search yielded 12 815 articles (12 262 from the original database search, 521 from the rapid update, and 32 from other sources; figure), of which 7817 remained after duplicates were removed. A further 6711 articles were removed after title and abstract screening. The full texts of the remaining 1106 articles were screened, including 47 articles that were translated into English for the purposes of this review: 13 from Spanish, 11 from German, six from French, four from Portuguese, three from Japanese, two from Chinese, two from Croatian, two from Italian, and one each from Danish, Dutch, Persian, and Russian. Of the full-text articles screened, 805 were excluded, leaving 301 articles: 260 primary research articles and 41 reviews. 56 (22%) primary research articles were excluded after assessment for quality (48 from high-income countries and eight from low-income and middle-income countries [LMICs]). The final review comprised 204 primary research articles and 41 reviews. Most primary research articles (183 [90%]) came from high-income countries and the remaining 21 (10%) came from LMICs. Findings are presented here, grouped into six key health domains: mental disorders (excluding substance use disorders), self-harm and suicidal behaviour, substance use and substance use disorders, neurodevelopmental disabilities, blood-borne viruses and STIs, and sexual and reproductive health.

### Mental disorders

90 publications, including 18 reviews, reported on mental disorders in detained adolescents (table 1). 47 (52%) were done in the USA and 18 (20%) in LMICs. Detained adolescents had a markedly higher prevalence of mental disorders than their community peers.^22,23^ One USA-based review of the health of detained adolescents^2^ reported that 66·8% of male and 81·0% of female adolescents met the diagnostic criteria for at least one mental disorder, with depression, behavioural disorders, and substance use disorders being the most prevalent. The reported point prevalence of any anxiety disorder in detained adolescents ranged from 3·4% to 31·5% for males (mean 17·4% [SD 8·1]; 17·8% [IQR 11·9–22·1])^24,25^ and from 20·9% to 59·0% in females (31·9% [11·6]; 30·3% [26·0–31·4]; appendix p 6).^24,26,27^ The reported point prevalence of post-traumatic stress disorder ranged from 0·0% to 53·0% for males (17·4% [14·1]; 14·1% [9·0–24·5])^28–30^ and from 13·0% to 65·1% for females (27·5% [17·2]; 20·0% [14·7–35·0]).^28,30,31^ Of the 14 papers that investigated psychotic disorders, the reported point prevalence of any psychotic disorder ranged from 0·8% to 2·0% for males (1·4% [0·6]; 1·3% [1·0–2·0])^22,26,32^ and from 1·0% to 9·0% for females (2·8% [3·1]; 1·5% [1·0–3·0]).^22,32,33^

Mood disorders were also highly prevalent among detained adolescents, with a reported point prevalence of any depressive disorder ranging from 4·0% to 36·0% for males (22·4% [14·2]; 26·2% [5·8–36·0])^29,31,32^ and from 14·0% to 63·0% for females (39·2% [16·8]; 33·3% [28·0–51·8]),^31,32,34^ and major depressive disorder ranging from 0·9% to 14·0% for males (9·1% [4·2]; 10·8% [6·7–11·4])^35–37^ and from 7·4% to 36·0% for females (24·2% [14·9]; 29·2% [7·4–36·0]; appendix p 7).

The reported point prevalence of conduct disorder ranged from 26·0% to 95·0% for males (66·9% [18·7]; 73·5% [53·9–79·8]),^28,35,38–40^ and from 17·0% to 91·0% for females (57·1% [22·0]; 53·8% [43·1–77·5]; appendix p 8),^26,41–44^ and the reported point prevalence of oppositional defiant disorder ranged from 8·0% to 51·0% for males (26·9% [17·1]; 19·3% [14·5–48·0]),^26,28,29,37,38^ and from 17·5% to 62·0% for females (38·4% [15·8]; 39·7% [25·0–46·4]; appendix p 8).^22,26–28,38,44^

### Self-harm and suicidal behaviour

56 articles, including four reviews, reported on suicidal ideation (n=36), self-harm or suicide attempt (n=27), and suicide deaths (n=7). Almost all original studies (n=50; 96%) came from eight high-income countries (the USA, Canada, the UK, Germany, Belgium, Russia, Australia, and New Zealand), with just six (11%) studies coming from LMICs (Sri Lanka, Iran, and Jordan; table 2). Five studies reported composite suicide risk scores consisting of suicide ideation and attempt.^35,44–47^ Two studies compared suicide rates between detained adolescents and their community peers,^6,48^ and one study compared rates of suicidal ideation between detained adolescents and their community peers.^49^ Few studies compared rates of suicidal behaviour between detained adolescents and their community peers.^50^ Overall, the prevalence of suicidal behaviour was markedly higher among detained adolescents than among adolescents in the general population.^51–56^ In detained adolescents, the prevalence of suicidal ideation ranged from 12·7% to 59·0% over the lifetime,^57–60^ 2·9–30·6% during the past month,^25,28,61–64^ 2·2–80·0% during the past 6 months,^47,65,66^ and 15·4–58·1% during the past year^67^ (appendix p 9);^68^ and the lifetime prevalence of suicide attempts ranged from 4·0% to 29·4% for males (mean 16·8% [SD 7·1]; median 17·3% [IQR 12·2–20·9])^15,40,41,54,59,61,62^ and from 20·8% to 51·1% for females (37·3% [10·6]; 39·8% [25·4–43·0]).40,41,59,65 For both sexes combined, the prevalence of suicide attempts was 1·9–6·6% during the past month and 13·3–35·0% during the past year (appendix p 10).^11,28,29,41,45,57–64,66,67,69–81^ The prevalence of suicidal behaviour during detention ranged from 4·6% to 22·9%,^11,67,70,81,82^ and increased to 6·0–27·5%^77,78^ following release from detention. The most commonly reported methods of self-harm were cutting (26–52%), poisoning (23·8–75%), and hanging or strangulation (9·5–67%; see appendix [p 11] for combined self-harm findings).^57,59,78^ Although suicide accounted for ≤1% of all deaths among adolescents in detention,^11,74,83^ the risk of suicide following release from detention is estimated to be two to nine times greater than that of their age-matched and sex-matched peers.^6,11,48,82,84^

### Substance use and substance use disorders

90 publications, including 12 reviews, reported on substance use. 80 (89%) publications came from high-income countries. A large proportion of detained adolescents reported using illicit substances within the past year,^12^ including cannabis,^64,85^ cocaine,^86^ amphetamines,^87^ heroin,^88^ hallucinogens,^86,88,89^ and inhalants,^86^ in addition to using alcohol^64,85,90^ and tobacco^91^ (table 3). Few studies measured the frequency of use or quantity of specific substances used. In studies that measured tobacco use, almost all detained adolescents reported lifetime use.^88,89,91,92^ Few studies used validated screening tools to measure tobacco use, and few informative comparisons could be made between detained and non-detained adolescents. The reported prevalence of lifetime substance use disorder ranged from 22% to 96% for detained adolescents, in contrast to 7–11% for adolescents in the general population (appendix p 12).^37,93^ The reported prevalence of lifetime injecting drug use among detained adolescents ranged from 0·1% to 55% (appendix p 12).^90,94^ Established risk factors for substance use—including maltreatment early in life, unstable and dysfunctional family environments, peer and family substance use, and brain injury—were more common among detained adolescents than their community peers.^95^

### Neurodevelopmental disabilities

58 publications, including 12 reviews, reported on neuro-developmental disorders. 45 (78%) came from high-income countries. The reported prevalence of various neurodevelopmental disabilities among detained adolescents was higher than that among their community peers (table 4). Reported rates of learning difficulties among detained adolescents ranged from 10% to 32%,^96–100^ reflecting varied definitions and assumptions necessitated by an inability to perform full diagnostic testing. However, these rates are considerably higher than those reported in general population studies (table 4).^101,102^ Similar findings were reported for communication impairments, with evidence suggesting that a majority of detained adolescents had some form of difficulty with language that significantly affected their day-to-day functioning.^103,104^ Experiences of traumatic brain injury were common among detained adolescents. One recent review suggested that 32–50% of detained adolescents had had a traumatic brain injury that resulted in loss of consciousness during their childhood, compared with 5–24% of adolescents in the general population.^13^

Rates of attention-deficit hyperactivity disorder (ADHD) in the general population of children and adolescents are estimated to be between 3% and 9%, with the prevalence in males approximately four times greater than that among females.^105^ In contrast, among individuals in detention, the prevalence of ADHD has been reported to range from 2·3% to 49·1% for males (mean 20·2% [SD 12·8]; median 17·6% [IQR 11·7–24·6])^9,22,25,26,30,32,35–38,79,106–109^ and from 6·0% to 48·2% for females (26·7% [12·7]; 21·7% [18·5–37·3]).^9,22,24,27,30,38,39,42,44,69^ Although some evidence indicates a higher prevalence of autism spectrum disorder among incarcerated young people than among the general population,^110^ previous studies have used selected samples, making prevalence difficult to establish.

The prevalence of fetal alcohol spectrum disorder (FASD) was also higher among detained adolescents than in the general population. Four Canadian studies documented a prevalence of 11–23% in detained adolescents,^111–114^ and an Australian study published in 2018 reported a prevalence of 36%.^115^ By contrast, in the general populations of high-income countries, 2–5% of children are estimated to be born with FASD.^116^ Each of the aforementioned studies from Australia and Canada reported an especially high prevalence among detained Indigenous adolescents (19–47%), which is reflective of wider health inequalities and disparities.^117^ The scarce research on FASD, which was restricted to studies from Canada and Australia, is indicative of the geographically uneven spread of studies of childhood neuro-developmental disabilities in general, with little evidence available from detained adolescents in LMICs.

### Blood-borne viruses and sexually-transmitted infections

66 publications, including 12 reviews,^1,2,15,118–126^ reported on blood-borne viruses and STIs in detained adolescents (table 5). 41 (76%) of the 54 original studies were done in the USA, with the remainder from Australia (n=3), Canada (n=2), Iran (n=2), Brazil (n=1), Bulgaria (n=1), Pakistan (n=1), Tanzania (n=1), Russian (n=1), and six nations in the eastern Caribbean (n=1). The prevalence data from these studies are presented in the appendix (p 13).

Detained adolescents had an increased prevalence of many communicable diseases, STIs, and associated risk-taking behaviours (eg, unprotected sex, sharing injecting equipment) compared with their community peers.^2,85,127,128^ 34 original studies reported chlamydia or gonorrhoea prevalence, 31 (91%) of which were done in the USA. Evidence on the prevalence of syphilis among detained adolescents was sparse, as syphilis is markedly less prevalent than chlamydia, and gonorrhoea and is less often the target of routine screening.^124,125^ We identified five studies that reported syphilis prevalence, seven studies on hepatitis B virus (HBV) surface antigen prevalence, 12 studies on hepatitis C virus (HCV) antibody prevalence, and 15 on HIV prevalence.

### Sexual and reproductive health

18 publications, including three reviews,^1,124,129^ reported on sexual and reproductive health outcomes (table 6). 13 (87%) primary studies came from the USA and all studies provided data about pregnancy among detained female adolescents. The reported proportion of detained female adolescents who had ever been pregnant ranged from 20% to 37%.^27,43,66,86,130–135^ Two studies reported that between 2% and 6% of detained females were currently pregnant,^14,124^ and one US study indicated that pregnancy was the focus of 1·1 health-care visits per detained female per month (range 0–6 visits).^136^ Another study reported that 11% of young female detainees had at least one child.^86^ Three studies reported that 22–31% of detained adolescent males had ever been responsible for a pregnancy.^132,134,137^ Seven papers documented the respondents’ reported age of sexual debut; six studies reported an average age of 12–13 years,^27,66,130–133^ while one reported a range from 8 years to 13 years of age.^137^ No corresponding normative data could be located for non-detained children and adolescents of this age. Three studies^27,66,131^ reported the prevalence of contraception use and showed lower frequencies of regular contraception use and of condom use during the most recent sexual encounter among detained adolescents than among the general population.^138^ Two studies reported on the prevalence of pelvic inflammatory disease among females, with estimates ranging from 3% to 12%.^66,130^ Three studies reported the prevalence of other genital or pelvic symptoms, including sores on the penis or pain during urination in males (38%), and dysmenorrhoea (68%) or an abnormal cervical screen (16%) in females.^130,135,137^

## Discussion

To our knowledge, this is the first attempt to synthesise evidence from a broad and diverse global literature examining the health of detained adolescents. Our findings show that detained adolescents commonly experience poor health across a range of physical and mental health domains, including mental disorders, self-harm and suicidal behaviour, substance use disorders, neuro-developmental disabilities, blood-borne viruses and STIs, and sexual and reproductive health. In studies that permitted a comparison with non-detained adolescents, adolescents in detention had consistently poorer health profiles. Although dominated by literature from high income countries (particularly the USA), the findings were broadly consistent across high-income, middle-income, and low-income countries and, when viewed from a public health perspective, present both challenges and opportunities.^139,140^

Many adolescents under-utilise primary and preventive care in the community before detention.^16^ Although this is true in high-income countries,^16^ no comparable data exist from low-income countries, although it is likely that high levels of unmet need also exist in such settings. Accordingly, detention often provides vulnerable adolescents with unique (yet regrettable) opportunities for diagnosis, disease management education, medical treatment, and counselling that they might otherwise not have accessed in the community.^140^ For example, our findings indicate that detained adolescents have a markedly higher prevalence of mental disorders^22,23^ and suicidal behaviours^6,48^ than their community peers. Most detained adolescents with mental disorders return to the community after release from detention, and poorer mental health is associated with higher rates of recidivism.^139^ As such, timely identification and subsequent provision of appropriate mental health care in adolescent detention settings has the potential to simultaneously improve mental health outcomes after release from detention and reduce rates of reincarceration. Detained adolescents with a history of suicidal behaviours are an especially at-risk group, with a high prevalence of mental and substance use disorders and social risk factors.^10^ They could benefit from targeted mental health interventions specifically designed to address impulsivity while in detention, as well as transitional mental health care and post-release support.

Targeted, evidence-based preventive efforts are urgently needed to address the health and social determinants of adolescent detention, and to provide timely health care to this highly marginalised population.^1^ For example, illicit substance use, by definition, involves illegal behaviours (ie, buying and possessing illicit drugs) which can increase the risk of contact with the criminal justice system and subsequent detention. In parallel with efforts to recognise substance use as a health issue rather than a criminal justice issue,^141^ increased access to developmentally appropriate harm reduction and drug treatment services in the community could simultaneously improve health outcomes and reduce criminal justice system contact among adolescents who engage in problematic substance use. Similar services should also be made available to adolescents in detention, with evidence indicating that motivational interviewing can be an effective intervention for reducing substance use in detained adolescents.^142^ The pharmacological effects of some substances, notably alcohol and amphetamines, can increase the likelihood of involvement in violent behaviour.^143^ Additionally, substance misuse can interfere with an adolescent’s successful transition to adult roles, including educational attainment and workforce participation, which can increase the likelihood of further detention.^144^ Recommendations to address harmful substance use embedded within detention settings include routine screening of all detained adolescents to identify harmful substance use and dependence as early as possible, provision of appropriate evidence-based harm reduction and drug treatment services, and comprehensive transitional support during re-entry into the community.^145^ In addition to measuring route of administration, important parameters to measure in substance use research with detained adolescents include frequency of use and quantity used, which are strongly correlated with drug-related harms. However, few studies in our Review included such data.

Our findings highlight a higher prevalence of several neurodevelopmental disabilities among detained adolescents when compared with their non-detained peers.^101–104^ Consideration must be given to the mechanisms by which cognitive, communicative, or socio-emotional difficulties associated with neurodevelopmental disabilities increase the risk of persistent offending and eventual detention.^146^ Insufficient awareness or assessment of neurodevelopmental disability can lead to a failure to understand important potential influences on antisocial behaviour or causes of poor engagement in interventions intended to address or reduce recidivism. Neurodevelopmental disability is also a risk factor for other health difficulties, including self-harm and substance misuse.^147^ Bespoke interventions supporting developmental needs that are well evidenced in other settings should also be employed within criminal justice settings such as adolescent detention facilities.^3^

Detention provides an opportunity to initiate treatment for myriad health conditions, such as catch-up vaccinations to protect against HBV.^148^ The wide variation in the seroprevalence of HBV and HCV observed in our Scoping Review probably reflects differences in both background prevalence, population immunisation policies, and criminal justice policies in different settings. Although a low prevalence of HBV and HCV among detained adolescents indicates a need for evidence-based prevention strategies, any non-zero prevalence indicates that detention facilities are important sites for diagnosis and treatment. Similarly, two reviews included in our Scoping Review^118,125^ documented that HIV infection was rare among detained adolescents in high-income countries, despite early sexual debut and unsafe sex being commonly reported. This finding highlights important opportunities for education and HIV prevention during detention for adolescents at increased risk of these outcomes.^118,125^ Detained adolescents are more likely than their non-detained peers to report an early age of sexual debut,^149^ and previous research has shown an association between early sexual debut and subsequent exposure to the criminal justice system,^150,151^ probably reflecting the shared social risks associated with these two outcomes. Routine screening for chlamydia and gonorrhoea for adolescents in detention is recommended by the US Centers for Disease Control and Prevention.^152^ The high prevalence of chlamydia among detained adolescents, as shown in our Scoping Review, underscores the importance of routine STI screening in detention facilities, which could also create opportunities for engagement around broader elements of sexual and reproductive health. Additionally, the high prevalence of pelvic inflammatory disease^66,130^ increases the risk of reproductive complications in this population in the future. Several studies have shown that high proportions of young detained females have experienced childhood sexual abuse or intimate partner violence,^77,153^ suggesting a need for trauma-informed approaches to sexual health (eg, allowing self-collected specimens rather than pairing STI screening with gynaecological examinations, and being able to request examination by a doctor of the same gender)^130^ in this population.

We documented a high prevalence of current (2–7%) and previous (20–37%) pregnancies in detained adolescent females and a high proportion of adolescent males who had fathered a child or been responsible for pregnancy (22–31%), all of whom were teenagers when they commenced their current detention. When considered in conjunction with the high rates of substance use disorders, including risky alcohol use, this high prevalence of pregnancy during adolescence increases the likelihood of intergenerational transmission of conditions such as FASD and perinatal substance dependence. Prevention efforts for such disorders should be focused on the most at-risk and disadvantaged groups in society (including detained adolescents, both males and females), and of increasing awareness of pregnancy and its prevention and providing access to condoms and other effective contraception, such as long-acting reversible contraceptives.

Addressing the unmet health-care needs of detained adolescents is an issue at the nexus of criminal justice reform and health-care reform.^2^ In light of our findings, efforts to better understand the physical and mental health trajectories of detained adolescents, and how these trajectories might be altered to improve morbidity outcomes and reduce mortality risk, should be considered an urgent priority. Such opportunities exist in research, clinical care, medical education, policy, and advocacy to drive improvements in the health of adolescents who have been detained. Diverting adolescents from detention and into treatment where appropriate, and addressing the health needs of those already detained, are crucial goals to protect adolescents and their families from further adverse health and social outcomes.^2^ Providing additional support to adolescents at increased risk of being exposed to the criminal justice system is likely to contribute to a reduction in the number of adolescents being detained. Furthermore, efforts to improve the health of adolescents at increased risk is likely to contribute to improvements in public health (because almost all incarcerated adolescents return to the community) and public safety (arising from the lower recidivism rates associated with improvements in health).^139^ These effects, in turn, will probably result in economic benefits by reducing the burden on both the health and criminal justice systems, and confer benefits for the next generation of at-risk children and adolescents.^154^

Our study also shows the limitations of the literature in this field. First, we identified large knowledge gaps relating to domains with significant ramifications for health, including asthma (no studies), rheumatic heart disease (no studies), and dental health (we identified a single cross-sectional study from Brazil examining the oral health of 102 detained male adolescents).^155^ Second, it is apparent that this is a relatively new area of research; all 241 included studies were published between 1980 and 2018, with 233 (97%) published since the year 2000. This recent increase in research on the health of detained adolescents is encouraging, but much work remains to be done. Third, most studies in our Scoping Review (90% of original research studies and 100% of reviews) came from high-income countries, with a majority from the USA. More robust, independent research examining the health of detained adolescents in LMICs is urgently needed. Fourth, more than one in five studies (22%) that met inclusion criteria were deemed to be of poor quality and were subsequently excluded. The 2016 *Lancet* Commission on Adolescent Health and Wellbeing^156^ called for the urgent collection of more high-quality data on the health of socially and economically marginalised adolescents, including those who come into contact with the criminal justice system. Similarly, the 2017 *Lancet* Inclusion Health series identified incarcerated young people as a particularly at-risk group, and called for more high-quality research on their health and wellbeing.^157,158^ Fifth, males made up a large proportion of all primary research study samples, while fewer data on the health of detained adolescent girls were available. Finally, we were unable to produce pooled regional or global prevalence estimates because of the large heterogeneity observed in study designs included in the Review.

Detained adolescents have poor health profiles across a variety of domains. Complex health needs in these adolescents are common and are often set against a backdrop of entrenched disadvantage. Many of the antecedents of poor health in this population are strongly linked to criminal justice involvement, such that policies regarding adolescent detention are relevant to health equity at the population level.^159^ More high-quality data, especially from LMICs, are urgently needed to inform targeted, evidence-based preventive strategies to address the social and structural drivers of adolescent detention and to provide timely health care to this highly marginalised group. Concurrent initiatives to reduce adolescent detention are crucial and must be made in parallel with proportionate investment in alternative ways of identifying and addressing their unmet health needs in the community. Efforts to better understand and improve the physical and mental health trajectories of detained adolescents, and how these trajectories might be altered to improve health and reduce mortality, will contribute to an improvement in broader public health.^139^ In the interim, greater investment in routine, comprehensive screening of all adolescents entering detention, coupled with evidence-based treatment in detention settings, will help to reduce the burden of preventable disease in these marginalised young people. As many of the health conditions experienced by detained adolescents are carried into the community^127^—with clear implications for population public health, appropriate access to transitional health care and social support needs to be continued in the community following release from detention to ensure that the improvements in health that are frequently reported during detention are not lost following release.

## Supplementary Material

Supplementary appendix

## Figures and Tables

**Figure:**
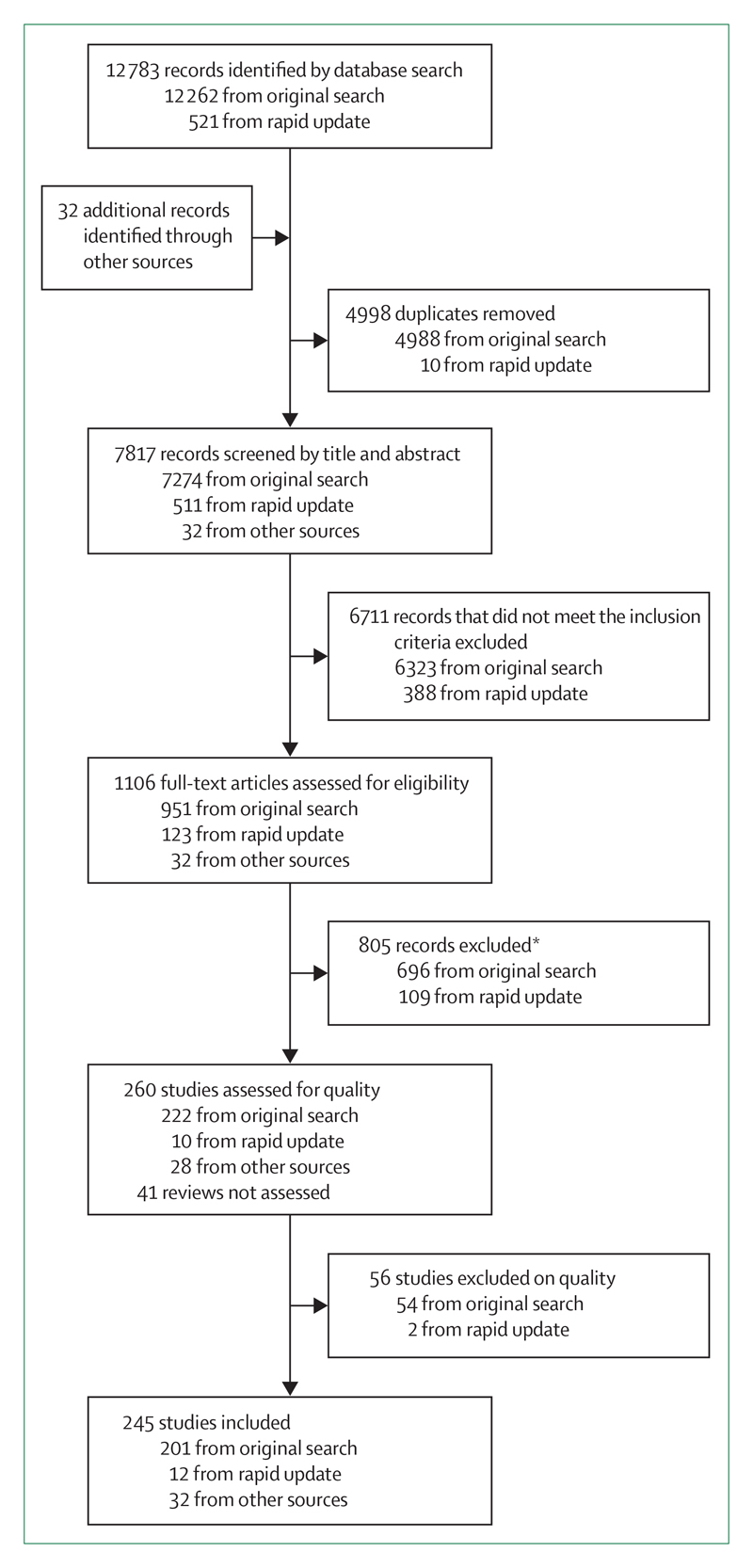
Study selection profile *Sample includes people ≥20 years of age (n=120), prevalence not reported or could not be determined (n=125), no outcome of interest reported (n=120), selected sample (n=164), sample not in youth justice detention or with no history of youth justice detention (n=145), sample includes people not in youth justice detention or without history of youth justice detention (n=72), poor ascertainment or definition of the outcome (n=28), not a journal article (n=18), self-reported delinquency (n=7), unable to confirm detention (n=3), full text not found (n=2), sample size too small (n=1).

**Table 1:** Prevalence of mental disorders in adolescents in detention and in the general population

	Point prevalence in detained adolescents, %*		Lifetime prevalence in detained adolescents, %	Lifetime prevalence in adolescents in the general population, %
	Males	Females		
Mood disorder (any)	10·1% (4·0–14·0); 0·4–36·0%^a1–a19^	26·6% (15·8–33·3); 0·0–63·0%^a2–a4,a6,a8,a9,a12,a13,a18,a20–a25^	7·0–82·0%^a6,a26–a29^	14·3%^a30^
Major depressive disorder	10·8% (6·7–11·4); 0·9–14·0%^a4–a7,a10,a11,a15,a16^	7·4–36·0%^a4,a6,a22^†	4·7–40·4%^a9,31–a35^	1·3%^a36^
Anxiety disorder (any)	17·8% (11·9–22·1); 3·4–31·5%^a2,3,a6,a13–a19,a23,a37^	30·3% (26·0–31·4); 20·9–59·0%^a2,a3,a6,a13,a22,a25,a27,a38^	9·0–56·3%^a5,a27,a29,a39,a40^	6·5%^a36^
Post-traumatic stress disorder	14·1% (9·0–24·5); 0·0–53·0%^a5–a8,a10,a11,a15,a16,a18,a23,a27,a37,a41–a47^	20·0% (14·7–35·0); 13·0–65·1%^a8,a20,a23,a27,a41–a43,a45^	11·0–48·9%^a9,a28,a33,a43^	5·0%^a30^
Obsessive-compulsive disorder	4·9% (2·6–7·0); 0·4–9·0%^a5,a6,a15,a16^	2·0–7·4%‡^a5,a6,a15,a16,a20,a35^	0·4–9·0%^a5,a6,a16,a20,a48^	0·3–4·0%^a49^
Conduct disorder	73·5% (53·9–79·8); 26·0–95·0%^a3–a8,a10–a12,a15–a17,a19,a34,a37,a41,a42,a50–a52^	53·8% (43·1–77·5); 17·0–91·0%^a3,a4,a6,a8,a12,a20–a22,a34,a41,a42,a53^	13·9–100%^a33,a40,a54–a56^	2·1%^a36^
Oppositional defiant disorder	19·3% (14·5–48·0); 8·0–51·0%^a3,a6,a8,a12,a16,a41,a57^	39·7% (25·0–46·4); 17·5–62·0%^a3,a6,a12,a21,a22,a41^	7·6–22·4%^a35,a39,a58,a59^	3·6%^a36^
Schizophrenia	2·0%; 0·8–2·2%^a5,a6,a51^§	1·9%^a6¶^	2·2–4·0%^a34,a60^	0·5–1·5%^a61^

For references see appendix (pp 15–29).

* Unless otherwise specified, data are median (IQR); range.

† Reported as range only, as n=3.

‡ Reported as range only, as n=2.

§ Reported as median; range only as n=3.

¶ Reported as point estimate only, as n=1.

**Table 2:** Prevalence of lifetime suicidal behaviour in adolescents in detention and in the general population

	Lifetime prevalence in detained adolescents (%)*	Lifetime prevalence in adolescents in the general population (%)
Suicidal ideation	..	15·3%^a69^
Males		
Lifetime	19·0% (14·1–24·7); 12·7–33·0%^a41,a62–a65^	..
Past month	8·6% (8·0–9·6); 7·0–11·6%^a15,a41,a66–a68^	..
Females		
Lifetime	38·3% (29·1–49·0); 21·6–58·0%^a41,a62–a64^	..
Past month	6·0–30·6%^a41,a68^†	..

Suicide attempt‡	..	4·1%^a75^
Males	17·3% (12·2–20·9); 4·0–29·4%^a15,a41,a42,a57,a62,a64,a65,a67,a68,a70–a72^	..
Females	39·8% (25·4–43); 20·8–51·1%^41,a42,a62,a68,a71,a73,a74^	..

Self-harm§	..	10·5–16·9%^a76,a77^
Males	20·9% (20·0–25·0); 12–34%^a41,a42,a57,a60,a71^	..
Females	47·1% (40·5–58·1); 38·0–65·0%^a41,a42,a60,a71^	..

Suicide¶	17·6–32%^a78,a79^	6·0–7·8%^a80,a81^ǁ

For references see appendix (pp 15–29).

* Unless otherwise specified, data are median (IQR); range.

† Reported as range only.

‡ Defined as a suicide attempt with intent to die.

§ Defined as deliberate self-harm and self-injurious behaviour.

¶ Proportion of overall mortality due to suicide.

ǁ Aggregated estimates for ages 10–24 years.^81^

**Table 3:** Prevalence of use of specific substances in adolescents in detention and in the general population

	Prevalence in detained adolescents (%)	Time period for past use*	Lifetime prevalence in adolescents in the general population (%)
Alcohol	50·9–90·1%^a13,a82–a85^	Past 1–12 months	6·0–45·0%^a30^

Cannabis	45·0–80·4%^a13,a82,a83,a86–a90^	Past 3 days to 12 months	6·0–42·0%^a91,a92^

Amphetamines (including methamphetamine)	8·2–25·8%^a85,a89,a93–a95^	Past 30 days to 3 months	0·0–12·0%^a92,a96^

Crack cocaine	1·5–15·2%^a88,a93,a97^	Past 1–4 months	0·7–2·7%^a92^

Cocaine (powdered or unspecified)	5·4–37·0%^a88,a89,a97,a98^	Past 3 days to 3 months	1·0–9·0%^a30,a96^

Heroin	1·0–6·5%^a86,a88,a97^	Past 3 days to 4 months	0·0–1·0%^a30,a96^

Inhalants	4·3–14·3%^a87,a89,a93,a97^	Past 4–6 months	8·0–11·0%^a92,a96^†

Any substance use disorder	..	..	7·0–11·0%^a30,a101,a102^
Males	50·7% (49·9–60); 11·0–85·5%^a6,a12,a41,a99,a100^§	NA	..
Females	59·4% (45·0–75); 12·0–100·0%^a6,a12,a41,a42,a99,a100^§	NA	..

Any substance use disorder (excluding alcohol use)†	9·4–60·0%^a10,a99,a100,a103–a106^	NA	4·3–5·5%^a102^

Alcohol use disorder	5·2–77·4%^a33,a48,a59,a103,a106–a109^	NA	4·6–6·0%^a102^

Cannabis use disorder	7·5–83·4%^a6,a12,a59,a103,a106,a109,a110^	NA	3·1–3·9%^a102^

For references see appendix (pp 15–29).

* Includes time before detention or time before interview.

† Includes opiate abuse and dependence.

‡ Includes amphetamine, cocaine, hallucinogen, inhalant, opiate, and sedative use disorders.

§ Reported as median (IQR); range.

**Table 4:** Prevalence of neurodevelopmental disabilities in adolescents in detention and in the general population

	Diagnostic criteria and typical symptoms	Reported prevalence in detained adolescents, %	Reported prevalence in adolescents in the general population, %
Attention-deficit hyperactivity disorder	Persistence in multiple symptoms of inattention, hyperactivity, and impulsivity	2–50%^a4,a111^	3–9%^a36,a112^
Communication impairments	Problems with speech, language, or hearing that significantly affect academic achievement or day-to-day social interactions; includes expressive and receptive language, speech sound disorder, and stuttering	60–65%^a113–a115^	5–7%^a113^
Fetal alcohol spectrum disorder	Reduced height, weight, or head circumference; characteristic facial features; deficits in executive functioning, memory, cognition, intelligence, attention, or motor skills; resulting from prenatal alcohol exposure due to maternal consumption during pregnancy	11–21%^a116–a120^	2–5%^a120^
Learning disability	Deficits in cognitive capacity (measured by an IQ score of <70); occasionally with adaptive functioning (significant difficulties with everyday tasks)	10–32%^a56,a121–a124^	2–4%^a125^
Traumatic brain injury	Disruption to the normal function of the brain resulting from a force to the head that causes loss of consciousness	32–50%^a126,a127^	15–20%^a128–a130^

For references see appendix (pp 15–29).

**Table 5:** Prevalence of blood-borne viruses and sexually transmitted infections in adolescents in detention and in the general population

	Prevalence in detained adolescents, %*	Reported prevalence in detained adolescents by study setting, %								Reported prevalence in adolescents in the general population (USA), %
		Australia	Brazil	Bulgaria	Canada	Iran	Russia	USA	Other countries
Chlamydia	..	..	..	..	..	..	..	8·3–12%^a159,a160^	..	2%^a161^
Males	8·2% (6·0–9·6); 2·0–14·4%^a131–a148^	2·0%^a148^	..	..	..	..	8·0%^a147^	4·8–14·4%^a131–a146^	..	..
Females	15·6% (13·3–23·3); 5·0–33·0%^a63,a73,a85,a133,a134,a136–a139,a142,a144,a145,a147–a157^	20%^a148^	..	..	10%^a152^	..	32%^a147^	5·0–33%^a63,a73,a85, a133–a139,a142,a144,a145, a149–a151,a154–a158^	..	..

Gonorrhoea	..	..	..	..	..	..	..	1·7–2·0%^a159,a160^	..	0·4%^a163^
Males	4·3% (1·5–6·6); 0·6–11·0%^a133,a134,a137,a139–a147,a162^	..	..	..	..	..	11%^a147^	0·6–6·7%^a133,a134,a137, a139–a146,a162^	..	..
Females	6·4% (5·6–16·0); 2·4–34·0%^a73,a89,a133,a134,a137,a139,a142,a144,a147,a149–a154,a162^	..	..	..	4%^a152^	..	34%^a147^	2·4–23·4%^a73,a133, a134,a137,a139,a142,a144,a145 ,a149–a151,a154,a158,a162^	..	..

Syphilis (antibody)	2·8% (0·9–3·4); 0·6–7·2%^a141,a147, a148,a162,a164^	3·0%^a148^	3·4%^a164^	..	..	..	7·2%^a147^	0·6–2·5%^a141,a162^	..	<0·01%^a165^

Hepatitis B virus (surface antigen)	0·7% (0·2–4·0); 0–25·3%^a126,a148,a164,a166–a169^	0·0–4·0%^a66,a148^	2·4%^a164^	25·3%^168^	..	0·6%^a166^	..	0·2–0·7%a167,^a169^	..	0·6%^a170^

Hepatitis C virus (antibody)	3·9% (2·1–9·9); 1–22%^a126,a148,a164, a167–a169,a171–a176^	9·0–10·8%^a66,a148^ (males 22%)^a176^	6·4%^a164^	20·4%^a168^	..	4·4%^a175^	..	1·0–3·4%^a167,a169, a171–a174^	..	<0·1%^a177^

HIV	0·3 (0·2–0·8); 0–2·2%^a66,a91,a147,a148, a164,a166,a168,a172,a178–a183^	0·0%^a66,a148^	0·3%^a164^	0·8%^a168^	0·3%^a180^	0·8%^a166^	1·9%^a147^	0·0–0·4%^a172,a178, a182,a183,a185^	Caribbean states (males) 2·2%^a179^; Pakistan 1·9%^a181^; Tanzania 2·1%^a184^	0·2%^a165^

For references see appendix (pp 15–29).

* Data are median (IQR); range.

**Table 6:** Prevalence of sexual and reproductive health outcomes in adolescents in detention and in the general population

	Detained adolescents	Adolescents in the general population
Age at sexual debut, years (range of mean age)	12·6–13·9^a22,a153,a186–a189^	16·0–17·0^a190,a191^
Ever pregnant (females only)	20·3–36·9%^a22,a53,a89,a153,a186–a189,a192,a193^	5·0–10·1%^a194^*^,a195^†
Currently pregnant (females only)	2·1–7·5%^a186,a196,a197^	0·1–5·7%^a198^‡
Fathered a child or responsible for a pregnancy (males only)	22·0–31·0%^a188,a192,a199^	··
Regular contraception use	66·1–79·3%^a22,a186^	89·9%^a200^§
Used condom during last sexual encounter	33·3%^a187^	53·8%^a201^
Ever had pelvic inflammatory disease	3·4–12·0%^a153,a186^	2·9%^a202^¶

For references see appendix (pp 15–29).

* Based on proportion of 15–20-year-old women reporting ever pregnant in population-based study in Switzerland.

† Based on proportion of 16–69-year-olds who reported a pregnancy at ≤20 years in population-based study in Australia.

‡ Based on annual adolescent pregnancy rate in high-income countries.

§ Any contraception use last sex (US national data).

¶ Prevalence of reported lifetime pelvic inflammatory disease among 18–24-year-olds (US national data).
