# Supplementary material for: The health of adolescents in detention: a global scoping review
Source: Lancet Public Health. Author manuscript; Available in PMC 2020 Feb 17. (PMC7025881; doi:10.1016/S2468-2667(19)30217-8)
Supplement: Supplementary appendix [file EMS85723-supplement-Supplementary_appendix.pdf]

THE LANCET
Public Health

Supplementary appendix

This appendix formed part of the original submission and has been peer reviewed. We post it as supplied by the authors.

Supplement to: Borschmann R, Janca E, Carter A, et al. The health of adolescents in detention: a global scoping review. *Lancet Public Health* 2020; published online Jan 16. https://doi.org/10.1016/S2468-2667(19)30217-8.

Appendix Reference List

Appendix 1a. PubMed search terms. 2

Appendix 1b. PubMed search strategy. 4

Figure 1. PRISMA flow chart of study selection 5

Anxiety disorders 6

Mood disorders 7

Behaviour disorders 8

Suicidal ideation 9

Suicide attempt 10

Self-harm 11

Substance use 12

Sexually transmitted infections 13

Infectious diseases 14

Appendix reference list 15

Appendix 1a. PubMed search terms.

Adolescence	Justice system	Juvenile offender	Health
Adolescent*	Detention	"detained adolescent"	Illness
Youth	"justice system"	"incarcerated adolescent"	Illnesses
Young	"correctional facility"	"incarcerated adolescents"	Disease
Juvenile*	"correctional facilities"	"juvenile offender"	Diseases
		"juvenile offenders"	Disorder
		"juvenile justice"	Disorders
		"juvenile detainee"	Infection
		"juvenile detainees"	Infections
		"juveniles detained"	Mortality
		"juvenile detention"	Mortalities
		"juveniles arrested"	Asthma
		"juvenile arrestee"	Suicide
		"juveniles arrested"	suicides
		"juvenile arrestees"	suicidal
		"incarcerated youth"	"self harm"
		"incarcerated youths"	"self destructive"
		"youth incarcerated"	"self mutilation"
		"detained youth"	"self inflicted"
		"detained youths"	"self injury"
		"young detainee"	"self injuries"
		"young detainees"	"self-harm"
		"young arrestee"	"self-destructive"
		"young arrestees"	"self-mutilation"
		"juvenile court"	"self-inflicted"
		"juvenile courts"	"self-injury"
		"young offender"	"self-injuries"
		"young offenders"	NSSI
		"juvenile delinquency"	"mental health problem"
		"juvenile delinquent"	"mental health problems"

		"juvenile delinquents"<br>"young delinquent"<br>"young delinquents"<br>"youth detained"<br><br>"young detained"<br>"young arrested"<br>"young incarcerated"	psychiatric<br>PTSD<br>ADHD<br>"oppositional defiant"<br>depression<br>anxiety<br>schizophrenia<br>bipolar<br>psychopathy<br>psychopathological<br>HIV<br>AIDS<br>STD<br>"STDs"<br>"sexually transmitted"<br>hepatitis<br>hepatic<br>"blood-borne"<br>TB<br>tuberculosis<br>herpes<br>"genital wart"<br>"genital warts"<br>pregnancy<br>"tobacco use"<br>smoking<br>"substance use"<br>"alcohol use"<br>"drug use"<br>"substance abuse"<br>"alcohol abuse"<br>"drug abuse"

			“substance misuse”<br>“alcohol misuse”<br>“drug misuse”<br>addiction<br>“risky sex”<br>“risky sexual”<br>“risky behaviour”<br>“risky behavior”<br>“risk behavior”<br>“risk behaviour”<br>“risky behaviours”<br>“risky behaviors”<br>“risk behaviors”<br>“risk behaviours”<br>“unsafe sex”<br>“unsafe sexual”

Appendix 1b. PubMed search strategy.

1. adolescen* OR young OR juvenile* OR youth*
2. “justice system” OR detention OR “correctional facility” OR “correctional facilities”
3. “incarcerated adolescents” OR “incarcerated adolescent” OR “juvenile offender” OR “juvenile offenders” OR “juvenile justice” OR “juvenile detainee” OR “juvenile detainees” OR “juveniles detained” OR “juvenile detention” OR “juveniles arrested” OR “juvenile arrestee” OR “juveniles arrested” OR “juvenile arrestees” OR “incarcerated youth” OR “incarcerated youths” OR “youth incarcerated” OR “detained youth” OR “detained youths” OR “young detainee” OR “young detainees” OR “young arrestee” OR “young arrestees” OR “juvenile court” OR “juvenile courts” OR “young offender” OR “young offenders” OR “juvenile delinquency” OR “juvenile delinquent” OR “juvenile delinquents” OR “young delinquent” OR “young delinquents” OR “youth detained” OR “young detained” OR “young arrested” OR “young incarcerated”
4. illness OR illnesses OR disease OR diseases OR disorder OR disorders OR infection OR infections OR mortality OR mortalities OR asthma OR suicide OR suicides OR suicidal OR “self harm” OR “self destructive” OR “self mutilation” OR “self inflicted” OR “self injury” OR “self injuries” OR “self-harm” OR “self-destructive” OR “self-mutilation” OR “self-inflicted” OR “self-injury” OR “self-injuries” OR NSSI OR “mental health problem” OR “mental health problems” OR psychiatric OR PTSD OR ADHD OR “oppositional defiant” OR depression OR anxiety OR schizophrenia OR bipolar OR psychopathy OR psychopathological OR HIV OR AIDS OR STD OR “STDs” OR “sexually transmitted” OR hepatitis OR hepatic OR “blood-borne” OR “blood borne” OR TB OR tuberculosis OR herpes OR “genital wart” OR “genital warts” OR pregnancy OR “tobacco use” OR smoking OR “substance use” OR “alcohol use” OR “drug use” OR “substance abuse” OR “alcohol abuse” OR “drug abuse” OR “substance misuse” OR “alcohol misuse” OR “drug misuse” OR addiction OR “risky sex” OR “risky sexual” OR “risky behaviour” OR “risky behavior” OR “risk behavior” OR “risk behaviour” OR “risky behaviours” OR “risky behaviors” OR “risk behaviors” OR “risk behaviours” OR “unsafe sex” OR “unsafe sexual”
5. 1 AND 2 AND 4
6. 2 AND 4
7. 5 OR 6
8. Limit 7 to yr = “1980 – Current”

Figure 1. PRISMA flow chart of study selection

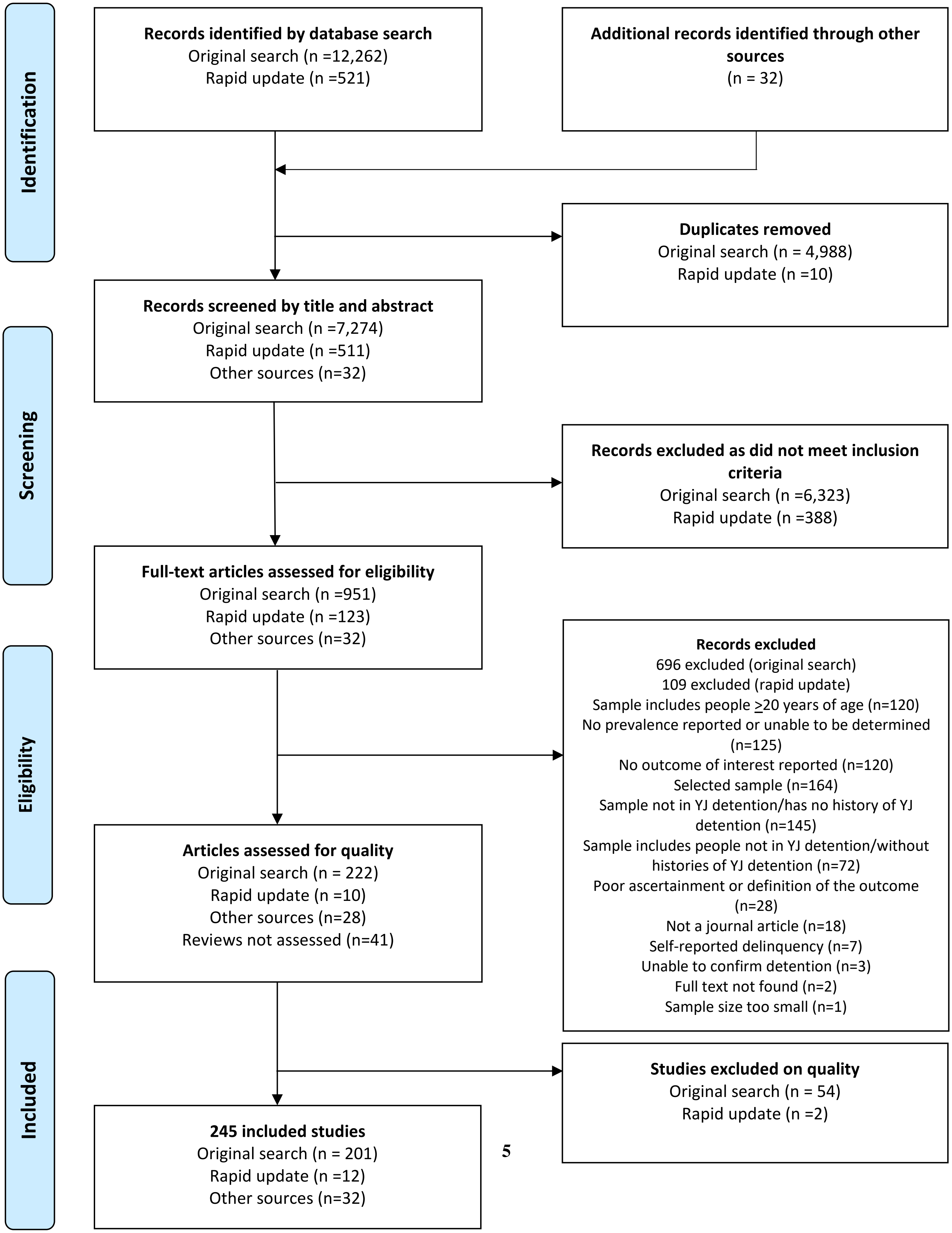

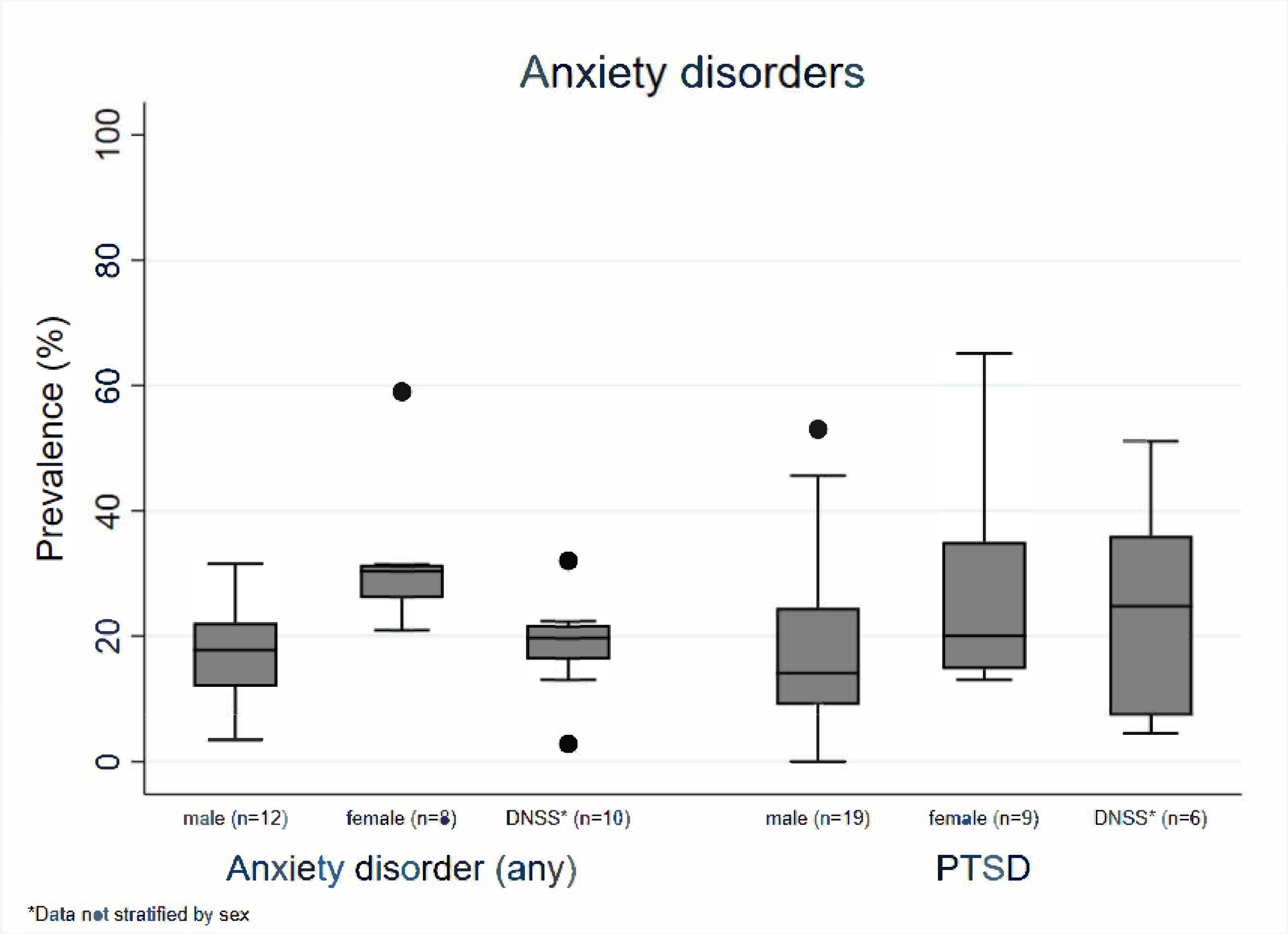
Anxiety disorders
Prevalence (%)
0
20
40
60
80
100
male (n=12)
female (n=8)
DNSS* (n=10)
male (n=19)
female (n=9)
DNSS* (n=6)
Anxiety disorder (any)
PTSD
*Data not stratified by sex

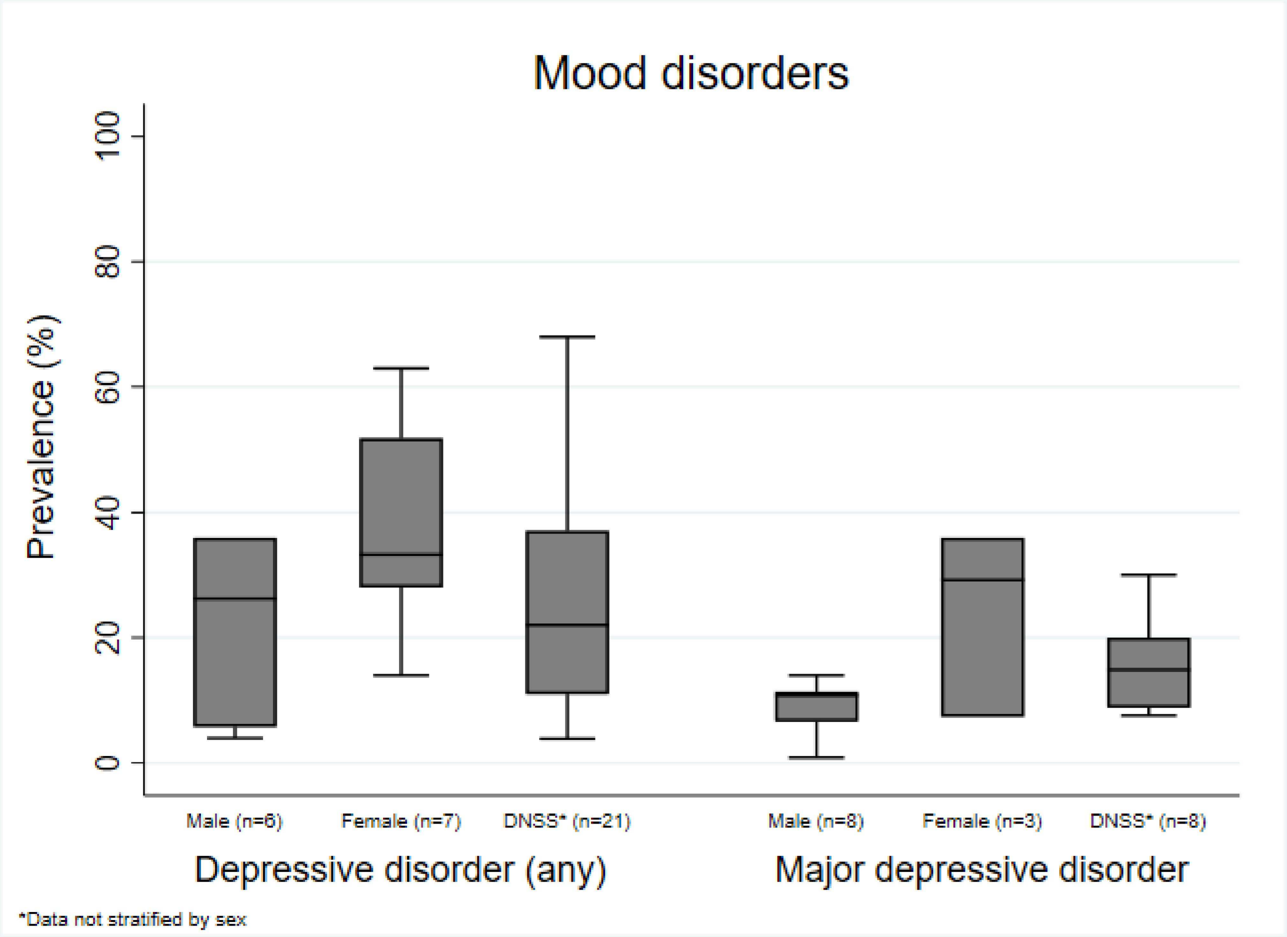
Mood disorders
Prevalence (%)
0
20
40
60
80
100
Male (n=6)
Female (n=7)
DNSS* (n=21)
Male (n=8)
Female (n=3)
DNSS* (n=8)
Depressive disorder (any)
Major depressive disorder
*Data not stratified by sex

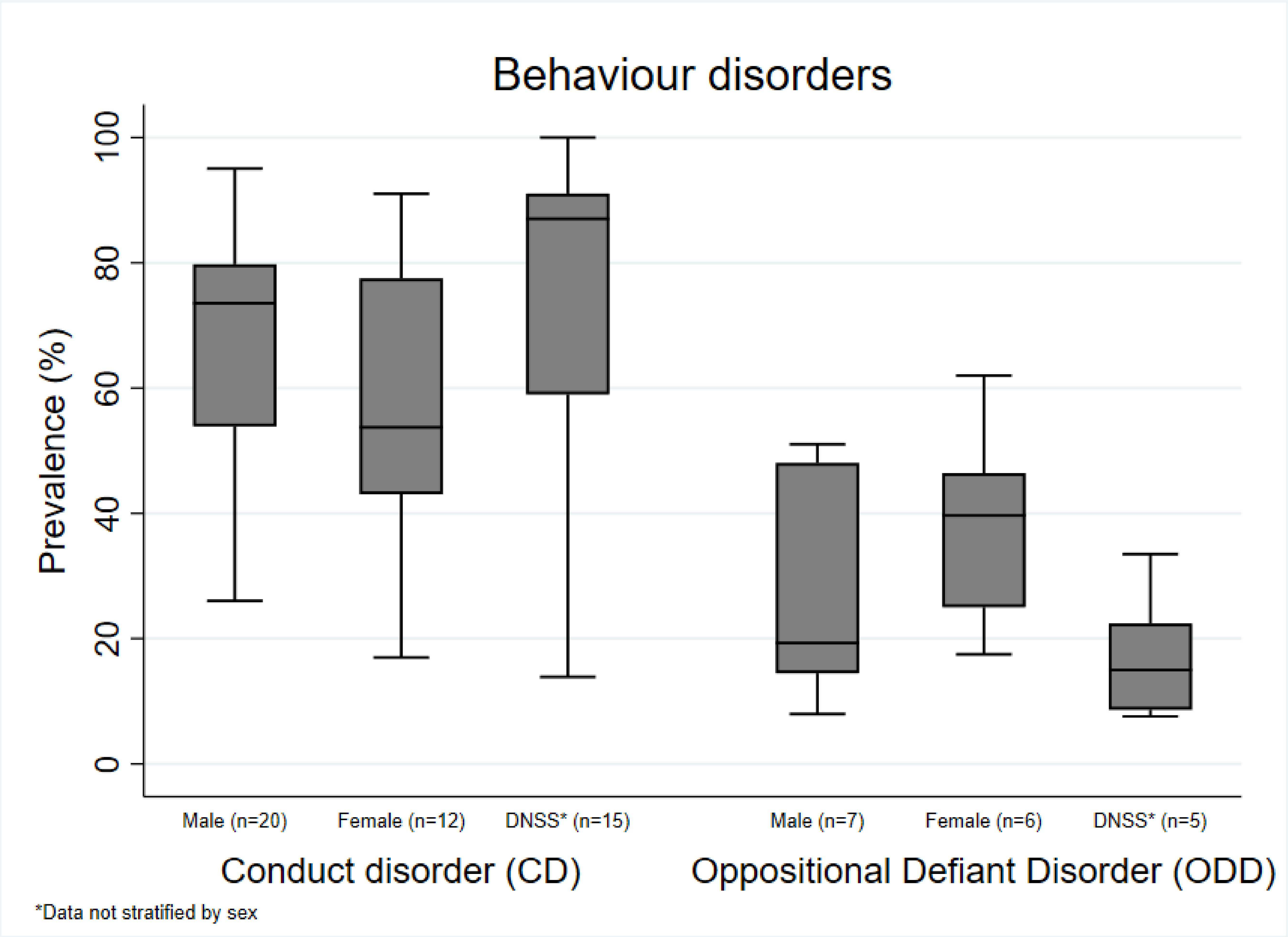
Behaviour disorders
Prevalence (%)
0
20
40
60
80
100
Male (n=20)
Female (n=12)
DNSS* (n=15)
Male (n=7)
Female (n=6)
DNSS* (n=5)
Conduct disorder (CD)
Oppositional Defiant Disorder (ODD)
*Data not stratified by sex

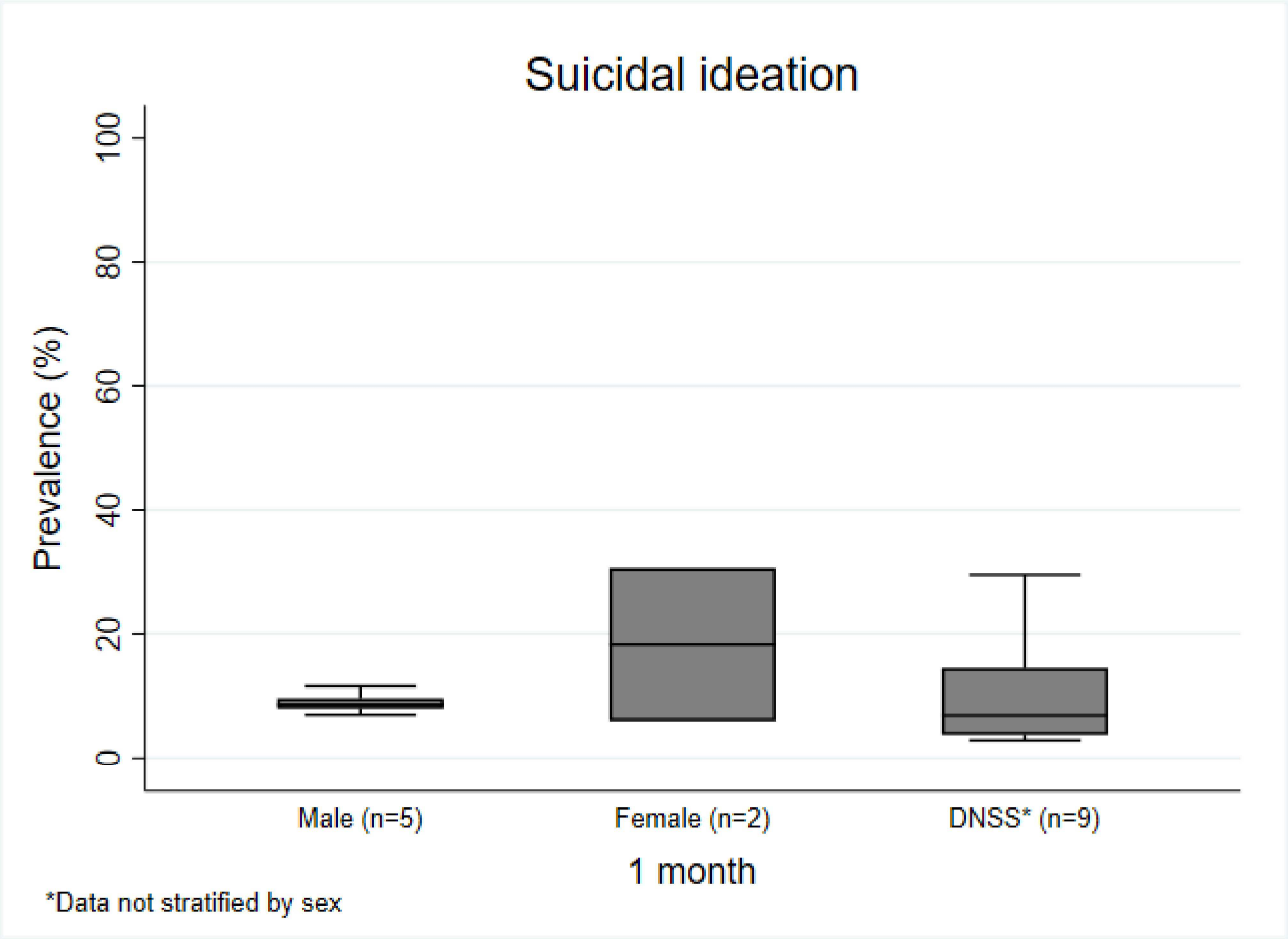
Suicidal ideation
Prevalence (%)
100
80
60
40
20
0
Male (n=5)
Female (n=2)
DNSS* (n=9)
1 month
*Data not stratified by sex

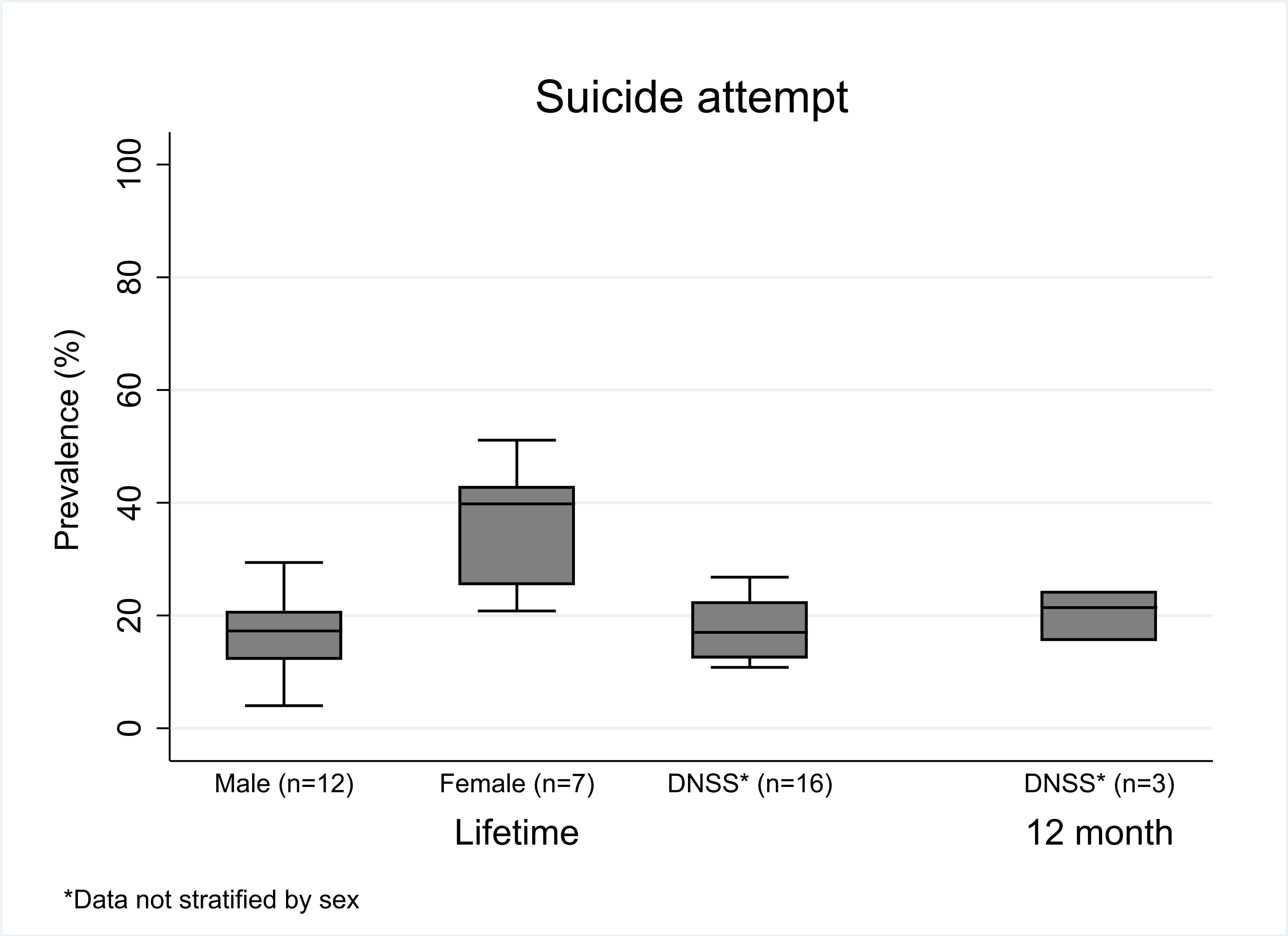
Suicide attempt
Prevalence (%)
100
80
60
40
20
0
Male (n=12)
Female (n=7)
DNSS* (n=16)
DNSS* (n=3)
Lifetime
12 month
*Data not stratified by sex

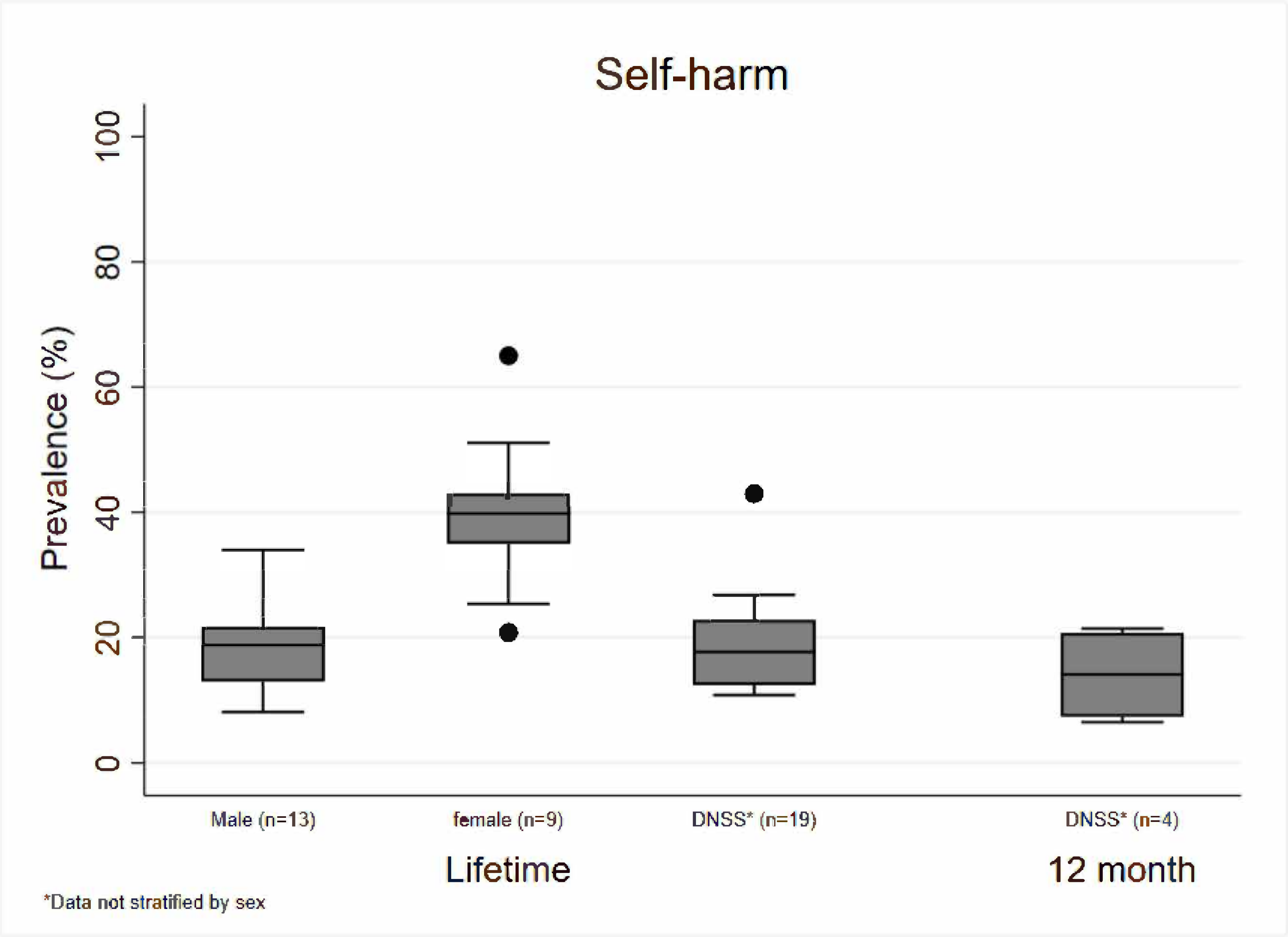
Self-harm
Prevalence (%)
0
20
40
60
80
100
Male (n=13)
female (n=9)
DNSS* (n=19)
DNSS* (n=4)
Lifetime
12 month
*Data not stratified by sex

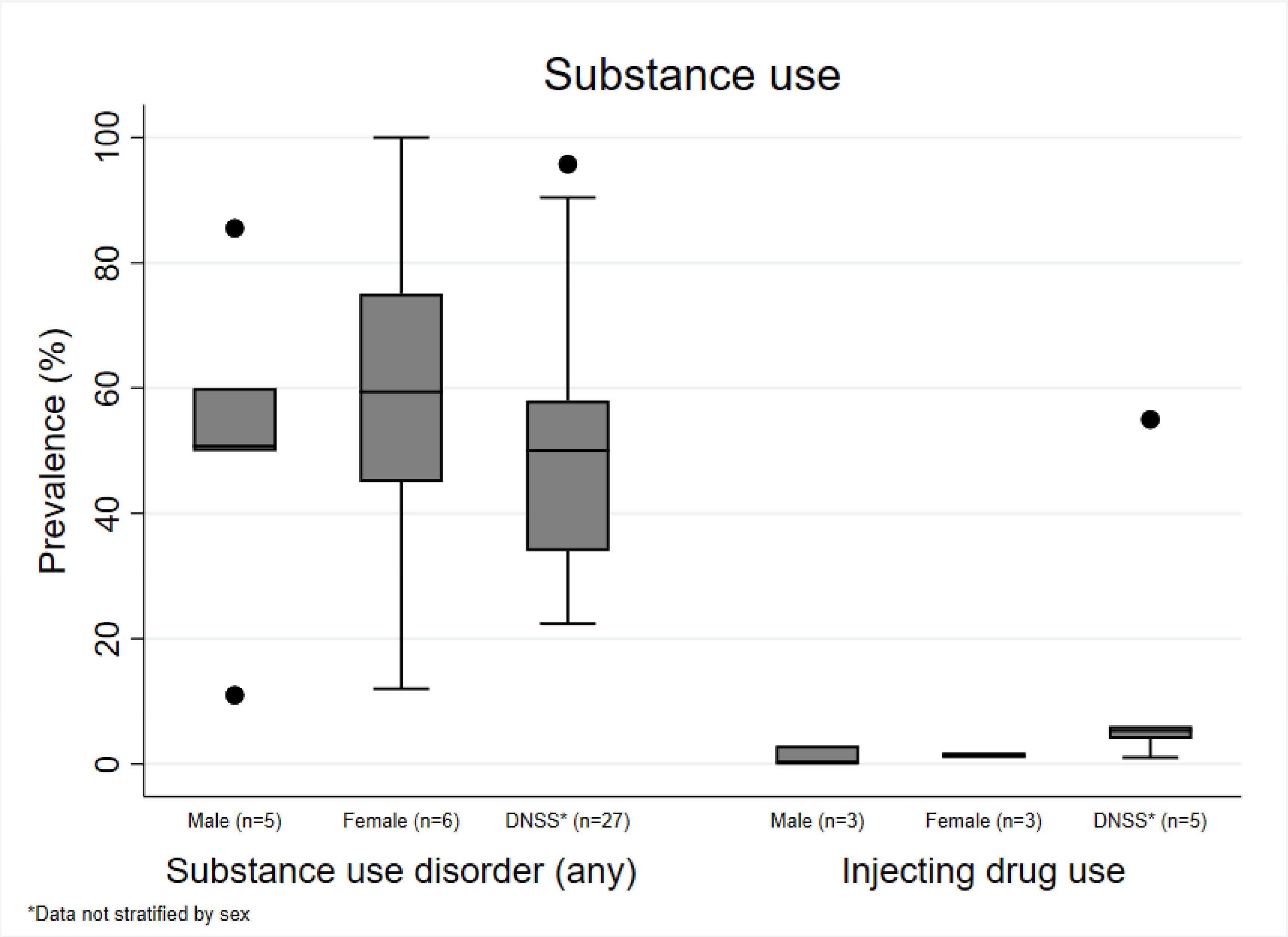
Substance use
Prevalence (%)
0
20
40
60
80
100
Male (n=5)
Female (n=6)
DNSS* (n=27)
Male (n=3)
Female (n=3)
DNSS* (n=5)
Substance use disorder (any)
Injecting drug use
*Data not stratified by sex

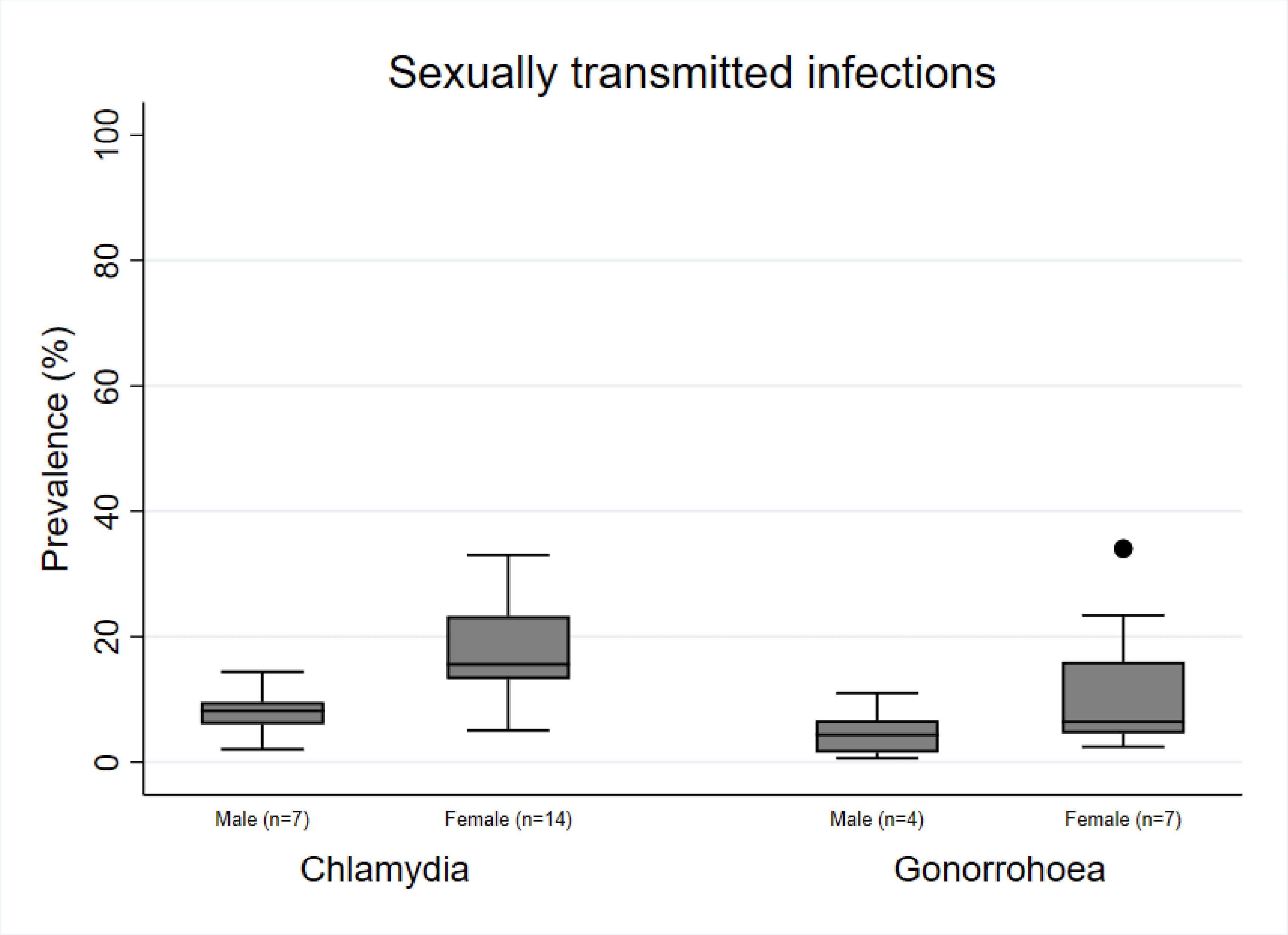

Sexually transmitted infections
Prevalence (%)
0
20
40
60
80
100
Male (n=7)
Female (n=14)
Male (n=4)
Female (n=7)
Chlamydia
Gonorrohoea

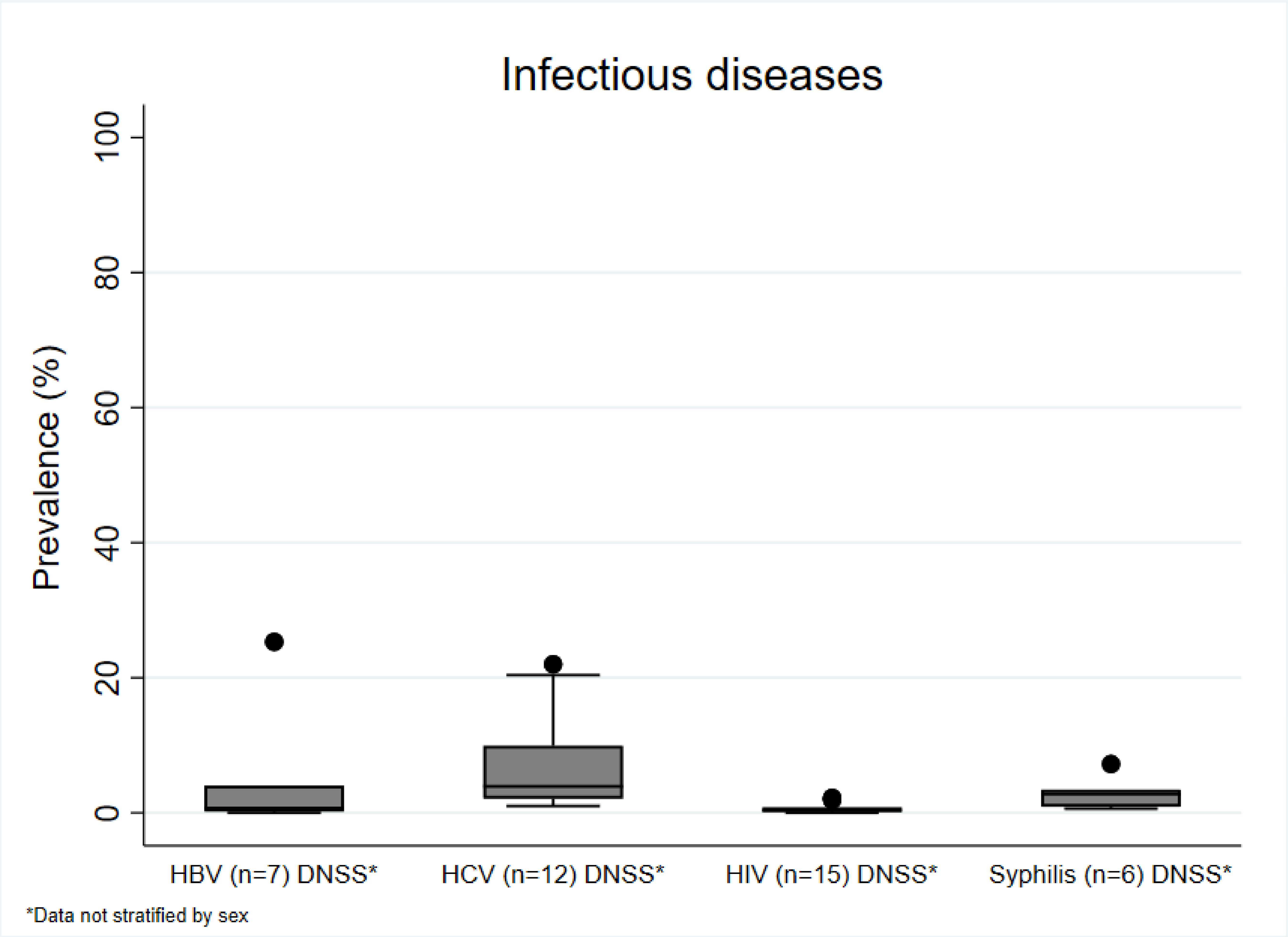
Infectious diseases
Prevalence (%)
100
80
60
40
20
0
HBV (n=7) DNSS*
HCV (n=12) DNSS*
HIV (n=15) DNSS*
Syphilis (n=6) DNSS*
*Data not stratified by sex

Appendix reference list.

a1 Abram KM, Choe JY, Washburn JJ, Teplin LA, King DC, Dulcan MK. Suicidal ideation and behaviors among youths in juvenile detention. *J Am Acad Child Adolesc Psychiatry* 2008; **47:** 291–300.

a2 Abram KM, Teplin LA, McClelland GM, Dulcan MK. Comorbid psychiatric disorders in youth in juvenile detention. *Arch Gen Psychiatry* 2003; **60:** 1097–108.

a3 Archer RP, Simonds-Bisbee EC, Spiegel DR, Handel RW, Elkins DE. Validity of the Massachusetts youth Screening Instrument-2 (MAYSI-2) scales in juvenile justice settings. *J Pers Assess* 2010; **92:** 337–48.

a4 Fazel S, Doll H, Långström N. Mental disorders among adolescents in juvenile detention and correctional facilities: a systematic review and metaregression analysis of 25 surveys. *J Am Acad Child Adolesc Psychiatry* 2008; **47:** 1010–19.

a5 Ghanizadeh A, Nouri SZ, Nabi SS. Psychiatric problems and suicidal behaviour in incarcerated adolescents in the Islamic Republic of Iran. *East Mediterr Health J* 2012; **18:** 311–17.

a6 Gretton HM, Clift RJ. The mental health needs of incarcerated youth in British Columbia, Canada. *Int J Law Psychiatry* 2011; **34:** 109–15.

a7 Koposov RA, Ruchkin VV, Eisemann M, Sidorov PI. Alcohol abuse in Russian delinquent adolescents. Associations with comorbid psychopathology, personality and parenting. *Eur Child Adolesc Psychiatry* 2005; **14:** 254–61.

a8 Lennings CJ, Kenny DT, Howard J, Arcuri A, Mackdacy L. The relationship between substance abuse and delinquency in female adolescents in Australia. *Psychiatry Psychol Law* 2007; **14:** 100–10.

a9 Lennox C. The health needs of young people in prison. *Br Med Bull* 2014; **112:** 17–25.

a10 Lindblad F, Isaksson J, Heiskala V, Koposov R, Ruchkin V. Comorbidity and behavior characteristics of Russian male juvenile delinquents with ADHD and conduct disorder. *J Atten Disord* 2015; published online April 29. DOI:10.1177/1087054715584052.

a11 Ruchkin V, Koposov R, Vermeiren R, Schwab-Stone M. Psychopathology and age at onset of conduct problems in juvenile delinquents. *J Clin Psychiatry* 2003; **64:** 913–20.

a12 Teplin LA, Abram KM, McClelland GM, Dulcan MK, Mericle AA. Psychiatric disorders in youth in juvenile detention. *Arch Gen Psychiatry* 2002; **59:** 1133–43.

a13 Teplin LA, Mericle AA, McClelland GM, Abram KM. HIV and AIDS risk behaviors in juvenile detainees: implications for public health policy. *Am J Public Health* 2003; **93:** 906–12.

a14 Vreugdenhil C, Vermeiren R, Wouters LF, Doreleijers TA, van den Brink W. Psychotic symptoms among male adolescent detainees in The Netherlands. *Schizophr Bull* 2004; **30:** 73–86.

a15 Wasserman GA, McReynolds LS, Lucas CP, Fisher P, Santos L. The voice DISC-IV with incarcerated male youths: prevalence of disorder. *J Am Acad Child Adolesc Psychiatry* 2002; **41:** 314–21.

a16 Zhou J, Chen C, Wang X, et al. Psychiatric disorders in adolescent boys in detention: a preliminary prevalence and case–control study in two Chinese provinces. *J Forensic Psychiatry Psychol* 2012; **23:** 664–75.

a17 Kongerslev M, Moran P, Bo S, Simonsen E. Screening for personality disorder in incarcerated adolescent boys: preliminary validation of an adolescent version of the Standardised Assessment of Personality–Abbreviated Scale (SAPAS-AV). *BMC Psychiatry* 2012; **12:** 94.

a18 Bielas H, Barra S, Skrivanek C, et al. The associations of cumulative adverse childhood experiences and irritability with mental disorders in detained male adolescent offenders. *Child Adolesc Psychiatry Ment Health* 2016; **10:** 34.

a19 Dória GM, Antoniuk SA, Assumpção Junior FB, Fajardo DN, Ehlke MN. Delinquency and association with behavioral disorders and substance abuse. *Rev Assoc Med Bras* 2015; **61:** 51–57.

a20 Dixon A, Howie P, Starling J. Psychopathology in female juvenile offenders. *J Child Psychol Psychiatry* 2004; **45:** 1150–58.

a21 Hamerlynck SM, Doreleijers TA, Vermeiren R, Jansen LM, Cohen-Kettenis PT. Aggression and psychopathology in detained adolescent females. *Psychiatry Res* 2008; **159:** 77–85.

a22 Lederman CS, Dakof GA, Larrea MA, Li H. Characteristics of adolescent females in juvenile detention. *Int J Law Psychiatry* 2004; **27:** 321–37.

a23 Abram KM, Washburn JJ, Teplin LA, Emanuel KM, Romero EG, McClelland GM. Posttraumatic stress disorder and psychiatric comorbidity among detained youths. *Psychiatr Serv* 2007; **58:** 1311–16.

a24 Goldstein NE, Arnold DH, Weil J, et al. Comorbid symptom patterns in female juvenile offenders. *Int J Law Psychiatry* 2003; **26:** 565–82.

a25 Kataoka SH, Zima BT, Dupre DA, Moreno KA, Yang X, McCracken JT. Mental health problems and service use among female juvenile offenders: their relationship to criminal history. *J Am Acad Child Adolesc Psychiatry* 2001; **40:** 549–55.

a26 Pyle N, Flower A, Fall AM, Williams J. Individual-level risk factors of incarcerated youth. *Remedial Spec Educ* 2016; **37:** 172–86.

a27 Gottfried ED, Christopher SC. Mental disorders among criminal offenders: a review of the literature. *J Correct Health Care* 2017; **23:** 336–46.

a28 Foy DW, Ritchie IK, Conway AH. Trauma exposure, posttraumatic stress, and comorbidities in female adolescent offenders: findings and implications from recent studies. *Eur J Psychotraumatol* 2012; **3:** 17247.

a29 Golzari M, Hunt SJ, Anoshiravani A. The health status of youth in juvenile detention facilities. *J Adolesc Health* 2006; **38:** 776–82.

a30 Merikangas KR, He JP, Burstein M, et al. Lifetime prevalence of mental disorders in U.S. adolescents: results from the National Comorbidity Survey Replication--Adolescent Supplement (NCS-A). *J Am Acad Child Adolesc Psychiatry* 2010; **49:** 980–89.

a31 Ryan EP, Redding RE. A review of mood disorders among juvenile offenders. *Psychiatr Serv* 2004; **55:** 1397–407.

a32 Kuo ES, Stoep AV, Stewart DG. Using the short mood and feelings questionnaire to detect depression in detained adolescents. *Assessment* 2005; **12:** 374–83.

a33 Ruchkin VV, Schwab-Stone M, Koposov R, Vermeiren R, Steiner H. Violence exposure, posttraumatic stress, and personality in juvenile delinquents. *J Am Acad Child Adolesc Psychiatry* 2002; **41:** 322–29.

a34 Singh Y, Kasinathan J, Kennedy A. Incarcerated youth mental and physical health: parity of esteem. *Int J Hum Rights Healthc* 2017; **10:** 203–12.

a35 Washburn JJ, Teplin LA, Voss LS, Simon CD, Abram KM, McClelland GM. Psychiatric disorders among detained youths: a comparison of youths processed in juvenile court and adult criminal court. *Psychiatr Serv* 2008; **59:** 965–73.

a36 Polanczyk GV, Salum GA, Sugaya LS, Caye A, Rohde LA. Annual research review: A meta-analysis of the worldwide prevalence of mental disorders in children and adolescents. *J Child Psychol Psychiatry* 2015; **56:** 345–65.

a37 Isaksson J, Grigorenko EL, Oreland L, Af Klinteberg B, Koposov RA, Ruchkin V. Exploring possible association between DβH genotype (C1021T), early onset of conduct disorder and psychopathic traits in juvenile delinquents. *Eur Arch Psychiatry Clin Neurosci* 2016; **266:** 771–73.

a38 Dixon A, Howie P, Starling J. Trauma exposure, posttraumatic stress, and psychiatric comorbidity in female juvenile offenders. *J Am Acad Child Adolesc Psychiatry* 2005; **44:** 798–806.

a39 Aida SA, Aili HH, Manveen KS, et al. Prevalence of psychiatric disorders among juvenile offenders in Malaysian prisons and association with socio-demographic and personal factors. *Int J Prison Health* 2014; **10:** 132–43.

a40 Bickel R, Campbell A. Mental health of adolescents in custody: the use of the “Adolescent Psychopathology Scale” in a Tasmanian context. *Aust N Z J Psychiatry* 2002; **36:** 603–09.

a41 Abrantes AM, Hoffmann NG, Anton R. Prevalence of co-occurring disorders among juveniles committed to detention centers. *Int J Offender Ther Comp Criminol* 2005; **49:** 179–93.

a42 Croysdale AE, Drerup LC, Bewsey K, Hoffmann NG. Correlates of victimization in a juvenile justice population. *J Aggress Maltreat Trauma* 2008; **17:** 103–17.

a43 Modrowski CA, Bennett DC, Chaplo SD, Kerig PK. Screening for PTSD among detained adolescents: Implications of the changes in the DSM-5. *Psychol Trauma* 2017; **9:** 10–17.

a44 Becker SP, Kerig PK. Posttraumatic stress symptoms are associated with the frequency and severity of delinquency among detained boys. *J Clin Child Adolesc Psychol* 2011; **40:** 765–71.

a45 Kerig PK, Bennett DC, Chaplo SD, Modrowski CA, McGee AB. Numbing of positive, negative, and general emotions: associations with trauma exposure, posttraumatic stress, and depressive symptoms among justice-involved youth. *J Trauma Stress* 2016; **29:** 111–19.

a46 Ruchkin VV, Koposov RA, af Klinteberg B, Oreland L, Grigorenko EL. Platelet MAO-B, personality, and psychopathology. *J Abnorm Psychol* 2005; **114:** 477–82.

a47 Stimmel MA, Cruise KR, Ford JD, Weiss RA. Trauma exposure, posttraumatic stress disorder symptomatology, and aggression in male juvenile offenders. *Psychol Trauma* 2014; **6:** 184–91.

a48 Wasserman GA, McReynolds LS, Lucas CP, Fisher P, Santos L. The voice DISC-IV with incarcerated male youths: prevalence of disorder. *J Am Acad Child Adolesc Psychiatry* 2002; **41:** 314–21.

a49 Krebs G, Heyman I. Obsessive-compulsive disorder in children and adolescents. *Arch Dis Child* 2015; **100:** 495–99.

a50 Dimond C, Misch P. Psychiatric morbidity in children remanded to prison custody—a pilot study. *J Adolesc* 2002; **25:** 681–89.

a51 Hollander HE, Turner FD. Characteristics of incarcerated delinquents: relationship between development disorders, environmental and family factors, and patterns of offense and recidivism. *J Am Acad Child Psychiatry* 1985; **24:** 221–26.

a52 Olashore AA, Ogunwale A, Adebowale TO. Correlates of conduct disorder among inmates of a Nigerian Borstal Institution. *Child Adolesc Psychiatry Ment Health* 2016; **10:** 13.

a53 Hamerlynck SM, Cohen-Kettenis PT, Vermeiren R, Jansen LM, Bezemer PD, Doreleijers TA. Sexual risk behavior and pregnancy in detained adolescent females: a study in Dutch detention centers. *Child Adolesc Psychiatry Ment Health* 2007; **1:** 4.

a54 DeLisi M, Neppl TK, Lohman BJ, Vaughn MG, Shook JJ. Early starters: Which type of criminal onset matters most for delinquent careers? *J Crim Justice* 2013; **41:** 12–17.

a55 Ollendick TH, Seligman LD, Butcher AT, M.Div. Does anxiety mitigate the behavioral expression of severe conduct disorder in delinquent youths? *J Anxiety Disord* 1999; **13:** 565–74.

a56 Haysom L, Indig D, Moore E, Gaskin C. Intellectual disability in young people in custody in New South Wales, Australia - prevalence and markers. *J Intellect Disabil Res* 2014; **58:** 1004–14.

a57 Ghanizadeh A, Nouri SZ, Nabi SS. Psychiatric problems and suicidal behaviour in incarcerated adolescents in the Islamic Republic of Iran. *East Mediterr Health J* 2012; **18:** 311–17.

a58 Ulzen T, Hamilton H. Post traumatic stress disorder in incarcerated adolescents. *Can Child Adolesc Psychiatr Rev* 2003; **12:** 113–16.

a59 Hoeve M, McReynolds LS, Wasserman GA. Comorbid internalizing and disruptive behavior disorder in adolescents: Offending, trauma, and clinical characteristics. *Crim Justice Behav* 2015; **42:** 840–55.

a60 Hettiarachchi LV, Kinner SA, Tibble H, Borschmann R. Self-harm among young people detained in the youth justice system in Sri Lanka. *Int J Environ Res Public Health* 2018; **15:** 209.

a61 APA. Diagnostic and statistical manual of mental disorders, 5th edn. Arlington, VA: American Psychiatric Association, 2013.

a62 Bhatta MP, Jefferis E, Kavadas A, Alemagno SA, Shaffer-King P. Suicidal behaviors among adolescents in juvenile detention: role of adverse life experiences. *PLoS One* 2014; **9:** e89408.

a63 Morris RE, Legault J, Baker C. Prevalence of isolated urethral asymptomatic Chlamydia trachomatis infection in the absence of cervical infection in incarcerated adolescent girls. *Sex Transm Dis* 1993; **20:** 198–200.

a64 Rohde P, Seeley JR, Mace DE. Correlates of suicidal behavior in a juvenile detention population. *Suicide Life Threat Behav* 1997; **27:** 164–75.

a65 Ruchkin V, Koposov RA, Koyanagi A, Stickley A. Suicidal behavior in juvenile delinquents: the role of ADHD and other comorbid psychiatric disorders. *Child Psychiatry Hum Dev* 2017; **48:** 691–98.

a66 Butler T, Belcher JM, Champion U, Kenny D, Allerton M, Fasher M. The physical health status of young Australian offenders. *Aust N Z J Public Health* 2008; **32:** 73–80.

a67 Kempton T, Forehand R. Suicide attempts among juvenile delinquents; the contribution of mental health factors. *Behav Res Ther* 1992; **30:** 537–41.

a68 Malvaso CG, Delfabbro PH, Day A, Nobes G. The maltreatment-violence link: Exploring the role of maltreatment experiences and other individual and social risk factors among young people who offend. *J Crim Justice* 2018; **55:** 35–45.

a69 Page RM, Saumweber J, Hall PC, Crookston BT, West JH. Multi-country, cross-national comparison of youth suicide ideation: findings from Global School-based Health Surveys. *Sch Psychol Int* 2013; **34:** 540–55.

a70 Aebi M, Bessler C, Plattner B. Autoaggression, external aggression and emotional problems in male juvenile detainees: Pilot study to validate an observational instrument for young prison inmates. *Forens Psychiatr Psychol Kriminol* 2017; **11:** 253–64 (in German).

a71 Freedenthal S, Vaughn MG, Jenson JM, Howard MO. Inhalant use and suicidality among incarcerated youth. *Drug Alcohol Depend* 2007; **90:** 81–88.

a72 Hales H, Davison S, Misch P, Taylor PJ. Young male prisoners in a young offenders' institution: their contact with suicidal behaviour by others. *J Adolesc* 2003; **26:** 667–85.

a73 Odgers CL, Robins SJ, Russell MA. Morbidity and mortality risk among the "forgotten few": why are girls in the justice system in such poor health? *Law Hum Behav* 2010; **34:** 429–44.

a74 Rohde P, Seeley JR, Mace DE. Correlates of suicidal behavior in a juvenile detention population. *Suicide Life Threat Behav* 1997; **27:** 164–75.

a75 Nock MK, Green JG, Hwang I, et al. Prevalence, correlates, and treatment of lifetime suicidal behavior among adolescents: results from the National Comorbidity Survey Replication Adolescent Supplement. *JAMA Psychiatry* 2013; **70:** 300–10.

a76 Gillies D, Christou MA, Dixon AC, et al. Prevalence and characteristics of self-harm in adolescents: meta-analyses of community-based studies 1990–2015. *J Am Acad Child Adolesc Psychiatry* 2018; **57:** 733–41.

a77 Kokkevi A, Rotsika V, Arapaki A, Richardson C. Adolescents' self-reported suicide attempts, self-harm thoughts and their correlates across 17 European countries. *J Child Psychol Psychiatry* 2012; **53:** 381–89.

a78 Coffey C, Veit F, Wolfe R, Cini E, Patton GC. Mortality in young offenders: retrospective cohort study. *BMJ* 2003; **326:** 1064.

a79 Gallagher CA, Dobrin A. Deaths in juvenile justice residential facilities. *J Adolesc Health* 2006; **38:** 662–68.

a80 Mokdad AH, Forouzanfar MH, Daoud F, et al. Global burden of diseases, injuries, and risk factors for young people's health during 1990–2013: a systematic analysis for the Global Burden of Disease Study 2013. *Lancet* 2016; **387:** 2383–401.

a81 Patton GC, Coffey C, Sawyer SM, et al. Global patterns of mortality in young people: a systematic analysis of population health data. *Lancet* 2009; **374:** 881–92.

a82 Romero EG, Teplin LA, McClelland GM, Abram KM, Welty LJ, Washburn JJ. A longitudinal study of the prevalence, development, and persistence of HIV/sexually transmitted infection risk behaviors in delinquent youth: implications for health care in the community. *Pediatrics* 2007; **119:** e1126–41.

a83 Putninš AL. Recent drug use and suicidal behaviour among young offenders. *Drug Alcohol Rev* 1995; **14:** 151–58.

a84 Tolou-Shams M, Stewart A, Fasciano J, Brown LK. A review of HIV prevention interventions for juvenile offenders. *J Pediatr Psychol* 2010; **35:** 250–61.

a85 McDonnell DD, Levy V, Morton TJ. Risk factors for chlamydia among young women in a northern California juvenile detention facility: implications for community intervention. *Sex Transm Dis* 2009; **36** (suppl)**:** S29–33.

a86 Yacoubian GS Jr, VanderWall KL, Johnson RJ, Urbach BJ, Peters RJ Jr. Comparing the validity of self-reported recent drug use between adult and juvenile arrestees. *J Psychoactive Drugs* 2003; **35:** 279–84.

a87 Hundleby JD. Personality and the prediction of delinquency and drug use: A follow-up study of school boys. *Br J Criminol* 1986; **26:** 129–46.

a88 Kang SY, Magura S, Shapiro JL. Correlates of cocaine/crack use among inner-city incarcerated adolescents. *Am J Drug Alcohol Abuse* 1994; **20:** 413–29.

a89 Robertson AA, Xu X, Stripling A. Adverse events and substance use among female adolescent offenders: effects of coping and family support. *Subst Use Misuse* 2010; **45:** 451–72.

a90 McKay J, Neal J. Diagnosis and disengagement: exploring the disjuncture between SEN policy and practice. *J Res Spec Educ Needs* 2009; **9:** 164–72.

a91 UN Office on Drugs and Crime. World drug report: drugs and age (booklet 4). Vienna: UN Office on Drugs and Crime, 2018.

a92 Johnston LD, O'Malley PM, Bachman JG, Schulenberg JE. Monitoring the future national survey results on drug use, 1975–2006: volume I—secondary school students. NIH Publication No. 06–5883. Bethesda, MD: National Institute on Drug Abuse, 2016.

a93 Braithwaite R, Robillard A, Woodring T, Stephens T, Arriola KJ. Tattooing and body piercing among adolescent detainees: relationship to alcohol and other drug use. *J Subst Abuse* 2001; **13:** 5–16.

a94 Yacoubian GS Jr, Wish ED, Choyka JD, Boyle CL, Harding CA, Loftus EA. Examining the prevalence and perceived harm of ecstasy and other drug use among juvenile offenders. *J Ethn Subst Abuse* 2004; **3:** 95–105.

a95 Burke C. Methamphetamine use among San Diego County arrestees. *J Psychoactive Drugs* 2007; **4** (suppl 4)**:** 337–45.

a96 Center for Behavioral Health Statistics and Quality. 2016 National Survey on Drug Use and Health: Detailed Tables. Rockville, MD: Substance Abuse and Mental Health Services Administration, 2017.

a97 McKay JR, Murphy RT, McGuire J, Rivinus TR, Maisto SA. Incarcerated adolescents' attributions for drug and alcohol use. *Addict Behav* 1992; **17:** 227–35.

a98 Yacoubian GS Jr, Arria AM, Fost E, Wish ED. Estimating the prevalence of Ecstasy use among juvenile offenders. *J Psychoactive Drugs* 2002; **34:** 209–13.

a99 Grigorenko EL, Edwards L, Chapman J. Cannabis use among juvenile detainees: typology, frequency and association. *Crim Behav Ment Health* 2015; **25:** 54–65.

a100 McClelland GM, Teplin LA, Abram KM, Jacobs N. HIV and AIDS risk behaviors among female jail detainees: implications for public health policy. *Am J Public Health* 2002; **92:** 818–25.

a101 Alegria M, Jackson JS, Kessler RC, Takeuchi D. Collaborative psychiatric epidemiology surveys (CPES), 2001–2003 [United States]. (ICPSR 20240). Ann Arbor, MI: Inter-university Consortium for Political and Social Research. March 23, 2016. https://www.icpsr.umich.edu/icpsrweb/ICPSR/studies/20240 (accessed Sept 10, 2019).

a102 Mericle AA, Arria AM, Meyers K, Cacciola J, Winters KC, Kirby K. National trends in adolescent substance use disorders and treatment availability: 2003–2010. *J Child Adolesc Subst Abuse* 2015; **24:** 255–63.

a103 Vreugdenhil C, Van Den Brink W, Wouters LF, Doreleijers TAH. Substance use, substance use disorders, and comorbidity patterns in a representative sample of incarcerated male Dutch adolescents. *J Nerv Ment Dis* 2003; **191:** 372–78.

a104 Johnson TP, Cho YI, Fendrich M, Graf I, Kelly-Wilson L, Pickup L. Treatment need and utilization among youth entering the juvenile corrections system. *J Subst Abuse Treat* 2004; **26:** 117–28.

a105 Teplin LA, Welty LJ, Abram KM, Dulcan MK, Washburn JJ. Prevalence and persistence of psychiatric disorders in youth after detention: a prospective longitudinal study. *Arch Gen Psychiatry* 2012; **69:** 1031–43.

a106 Pliszka SR, Sherman JO, Barrow MV, Irick S. Affective disorder in juvenile offenders: A preliminary study. *Am J Psychiatry* 2000; **157:** 130–32.

a107 Elkington KS, Teplin LA, Mericle AA, Welty LJ, Romero EG, Abram KM. HIV/sexually transmitted infection risk behaviors in delinquent youth with psychiatric disorders: a longitudinal study. *J Am Acad Child Adolesc Psychiatry* 2008; **47:** 901–11.

a108 Teplin LA, Jakubowski JA, Abram KM, Olson ND, Stokes ML, Welty LJ. Firearm homicide and other causes of death in delinquents: a 16-year prospective study. *Pediatrics* 2014; **134:** 63–73.

a109 Welty LJ, Harrison AJ, Abram KM, et al. Health disparities in drug- and alcohol-use disorders: a 12-year longitudinal study of youths after detention. *Am J Public Health* 2016; **106:** 872–80.

a110 Indig D, Frewen A, Moore E. Predictors and correlates of re-incarceration among Australian young people in custody. *Aust N Z J Criminol* 2016; **49:** 73–89.

a111 Young S, Moss D, Sedgwick O, Fridman M, Hodgkins P. A meta-analysis of the prevalence of attention deficit hyperactivity disorder in incarcerated populations. *Psychol Med* 2015; **45:** 247–58.

a112 National Collaborating Centre for Mental Health. Attention deficit hyperactivity disorder: diagnosis and management of ADHD in children, young people and adults. Leicester: British Psychological Society, 2009.

a113 Tomblin JB, Zhang X, Buckwalter P, Catts H. The association of reading disability, behavioral disorders, and language impairment among second-grade children. *J Child Psychol Psychiatry* 2000; **41:** 473–82.

a114 Gregory J, Bryan K. Speech and language therapy intervention with a group of persistent and prolific young offenders in a non-custodial setting with previously undiagnosed speech, language and communication difficulties. *Int J Lang Commun Disord* 2011; **46:** 202—15.

a115 Bryan K. Preliminary study of the prevalence of speech and language difficulties in young offenders. *Int J Lang Commun Disord* 2004; **39:** 391–400.

a116 Murphy A, Chittendon M. Time out II: a profile of BC youth in custody. Vancouver, BC: The McCreary Centre Society, 2005.

a117 Fast DK, Conry J, Loock CA. Identifying fetal alcohol syndrome among youth in the criminal justice system. *J Dev Behav Pediatr* 1999; **20:** 370–72.

a118 Rojas EY, Gretton HM. Background, offence characteristics, and criminal outcomes of Aboriginal youth who sexually offend: a closer look at Aboriginal youth intervention needs. *Sex Abuse* 2007; **19:** 257–83.

a119 Smith A, Cox K, Poon C, Stewart D. Time out III: a profile of BC youth in custody. Vancouver, BC: The McCreary Centre Society, 2013.

a120 May PA, Gossage JP, Kalberg WO, et al. Prevalence and epidemiologic characteristics of FASD from various research methods with an emphasis on recent in-school studies. *Dev Disabil Res Rev* 2009; **15:** 176–92.

a121 Kroll L, Rothwell J, Bradley D, Shah P, Bailey S, Harrington RC. Mental health needs of boys in secure care for serious or persistent offending: a prospective, longitudinal study. *Lancet* 2002; **359:** 1975–79.

a122 Chitsabesan P, Kroll L, Bailey S, et al. Mental health needs of young offenders in custody and in the community. *Br J Psychiatry* 2006; **188:** 534–40.

a123 Herrington V. Assessing the prevalence of intellectual disability among young male prisoners. *J Intellect Disabil Res* 2009; **53:** 397–410.

a124 Allerton M, Champion U, Kenny D, Butler T. 2003 NSW Young People in Custody Health Survey: a summary of some key findings. Juvenile Justice: from Lessons of the Past to a Road Map for the Future conference; Sydney, Australia; Dec 1–2, 2003.

a125 AIHW. Disability prevalence and trends. Canberra: Australian Institute of Health and Welfare, 2003.

a126 Butler T, Belcher JM, Champion U, Kenny D, Allerton M, Fasher M. The physical health status of young Australian offenders. *Aust N Z J Public Health* 2008; **32:** 73–80.

a127 Hughes N, Williams WH, Chitsabesan P, Walesby RC, Mounce LT, Clasby B. The prevalence of traumatic brain injury among young offenders in custody: a systematic review. *J Head Trauma Rehabil* 2015; **30:** 94–105.

a128 Segalowitz SJ, Brown D. Mild head injury as a source of developmental disabilities. *J Learn Disabil* 1991; **24:** 551–59.

a129 Segalowitz SJ, Lawson S. Subtle symptoms associated with self-reported mild head injury. *J Learn Disabil* 1995; **28:** 309–19.

a130 Ilie G, Boak A, Adlaf EM, Asbridge M, Cusimano MD. Prevalence and correlates of traumatic brain injuries among adolescents. *JAMA* 2013; **309:** 2550–52.

a131 Blake DRG, Gaydos CA, Quinn TC. Cost-effectiveness analysis of screening adolescent males for Chlamydia on admission to detention. *Sex Transm Dis* 2004; **31:** 85–95.

a132 Bortot ATR, Risser WL, Cromwell PF. Condom use in incarcerated adolescent males: knowledge and practice. *Sex Transm Dis* 2006; **33:** 2–4.

a133 Broussard DL, Leichliter JS, Evans A, Kee R, Vallury V, Mcfarlane MM. J. S.; Evans, A.; Kee, R.; Vallury, V.; McFarlane, M. M. Screening adolescents in a juvenile detention center for gonorrhea and chlamydia: Prevalence and reinfection rates. *Prison J* 2002; **82:** 8–18.

a134 Canterbury RJ, McGarvey EL, Sheldon-Keller AE, Waite D, Reams P, Koopman C. Prevalence of HIV-related risk behaviors and STDs among incarcerated adolescents. *J Adolesc Health* 1995; **17:** 173–77.

a135 Chartier M, Packel L, Bauer HM, Brammeier M, Little M, Bolan G. Chlamydia prevalence among adolescent females and males in juvenile detention facilities in California. *J Correct Health Care* 2004; **11:** 79–97.

a136 Joesoef MRW, Weinstock HS, Kent CK, et al. Sex and age correlates of Chlamydia prevalence in adolescents and adults entering correctional facilities, 2005: implications for screening policy. *Sex Transm Dis* 2009; **36** (suppl)**:** S67–71.

a137 Kahn RH, Mosure DJ, Blank S, et al. Chlamydia trachomatis and Neisseria gonorrhoeae prevalence and coinfection in adolescents entering selected US juvenile detention centers, 1997–2002. *Sex Transm Dis* 2005; **32:** 255–59.

a138 Kelly PJB, Bair RM, Baillargeon J, German V. Risk behaviors and the prevalence of Chlamydia in a juvenile detention facility. *Clin Pediatr (Phila)* 2000; **39:** 521–27.

a139 Mertz KJ, Voigt RA, Hutchins K, Levine WC. Findings from STD screening of adolescents and adults entering corrections facilities: implications for STD control strategies. *Sex Transm Dis* 2002; **29:** 834–39.

a140 O'Brien SF, Bell TA, Farrow JA. Use of a leukocyte esterase dipstick to detect Chlamydia trachomatis and Neisseria gonorrhoeae urethritis in asymptomatic adolescent male detainees. *Am J Public Health* 1988; **78:** 1583–84.

a141 Oh MK, Cloud GA, Wallace LS, Reynolds J, Sturdevant M, Feinstein RA. Sexual behavior and sexually transmitted diseases among male adolescents in detention. *Sex Transm Dis* 1994; **21:** 127–32.

a142 Oh MK, Smith KR, O'Cain M, Kilmer D, Johnson J, Hook EW 3rd. Urine-based screening of adolescents in detention to guide treatment for gonococcal and chlamydial infections. Translating research into intervention. *Arch Pediatr Adolesc Med* 1998; **152:** 52–56.

a143 Pack RP, Diclemente RJ, Hook EW 3rd, Oh MK. High prevalence of asymptomatic STDs in incarcerated minority male youth: a case for screening. *Sex Transm Dis* 2000; **27:** 175–77.

a144 Risser JM, Risser WL, Gefter LR, Brandstetter DM, Cromwell PF. Implementation of a screening program for chlamydial infection in incarcerated adolescents. *Sex Transm Dis* 2001; **28:** 43–46.

a145 Robertson AA, Thomas CB, St Lawrence JS, Pack R. Predictors of infection with Chlamydia or gonorrhea in incarcerated adolescents. *Sex Transm Dis* 2005; **32:** 115–22.

a146 Shafer M-A, Hilton JF, Ekstrand M, et al. Relationship between drug use and sexual behaviors and the occurrence of sexually transmitted diseases among high-risk male youth. *Sex Transm Dis* 1993; **20:** 307–13.

a147 Shakarishvili A, Dubovskaya LK, Zohrabyan LS, et al. Sex work, drug use, HIV infection, and spread of sexually transmitted infections in Moscow, Russian Federation. *Lancet* 2005; **366:** 57–60.

a148 Watkins RE, Mak DB, Connelly C. Testing for sexually transmitted infections and blood borne viruses on admission to Western Australian prisons. *BMC Public Health* 2009; **9:** 385.

a149 Bell TA, Farrow JA, Stamm WE, Critchlow CW, Holmes KK. Sexually transmitted diseases in females in a juvenile detention center. *Sex Transm Dis* 1985; **12:** 140–44.

a150 Cromwell PFR, Risser WL, Risser JM. Prevalence and incidence of pelvic inflammatory disease in incarcerated adolescents. *Sex Transm Dis* 2002; **29:** 391–96.

a151 Crosby R, Salazar LF, DiClemente RJ, Yarber WL, Caliendo AM, Staples-Horne M. Condom misuse among adjudicated girls: associations with laboratory-confirmed chlamydia and gonorrhea. *J Pediatr Adolesc Gynecol* 2007; **20:** 339–43.

a152 Gander S, Scholten V, Osswald I, Sutton M, van Wylick R. Cervical dysplasia and associated risk factors in a juvenile detainee population. *J Pediatr Adolesc Gynecol* 2009; **22:** 351–55.

a153 Holland-Hall CM, Wiesenfeld HC, Murray PJ. Self-collected vaginal swabs for the detection of multiple sexually transmitted infections in adolescent girls. *J Pediatr Adolesc Gynecol* 2002; **15:** 307–13.

a154 Javanbakht M, Boudov M, Anderson LJ, et al. Sexually transmitted infections among incarcerated women: findings from a decade of screening in a Los Angeles County Jail, 2002–2012. *Am J Public Health* 2014; **104:** e103–09.

a155 Katz ARL, Lee MV, Ohye RG, Effler PV, Johnson EC, Nishi SM. Prevalence of chlamydial and gonorrheal infections among females in a juvenile detention facility, Honolulu, Hawaii. *J Community Health* 2004; **29:** 265–69.

a156 Lofy KH, Hofmann J, Mosure DJ, Fine DN, Marrazzo JM. Chlamydial infections among female adolescents screened in juvenile detention centers in Washington State, 1998–2002. *Sex Transm Dis* 2006; **33:** 63–67.

a157 Salazar LF, Crosby RA, Diclemente RJ. Exploring the mediating mechanism between gender-based violence and biologically confirmed Chlamydia among detained adolescent girls. *Violence Against Women* 2009; **15:** 258–75.

a158 Holland-Hall CMW, Wiesenfeld HC, Murray PJ. Self-collected vaginal swabs for the detection of multiple sexually transmitted infections in adolescent girls. *J Pediatr Adolesc Gynecol* 2002; **15:** 307–13.

a159 Bryan AD, Magnan RE, Gillman AS, et al. Effect of including alcohol and cannabis content in a sexual risk-reduction intervention on the incidence of sexually transmitted infections in adolescents a cluster randomized clinical trial. *JAMA Pediatr* 2018; **172:** e175621.

a160 Crosby R, Voisin D, Salazar LF, DiClemente RJ, Yarber WL, Caliendo AM. Family influences and biologically confirmed sexually transmitted infections among detained adolescents. *Am J Orthopsychiatry* 2006; **76:** 389–94.

a161 Barnert ES, Perry R, Morris RE. Juvenile incarceration and health. *Acad Pediatr* 2016; **16:** 99–109.

a162 Alexander-Rodriguez T, Vermund SH. Gonorrhea and syphilis in incarcerated urban adolescents: prevalence and physical signs. *Pediatrics* 1987; **80:** 561–64.

a163 Aral SO, Fenton KA, Holmes KK. Sexually transmitted diseases in the USA: temporal trends. *Sex Transm Infect* 2007; **83:** 257–66.

a164 Fialho M, Messias M, Page-Shafer K, et al. Prevalence and risk of blood-borne and sexually transmitted viral infections in incarcerated youth in Salvador, Brazil: opportunity and obligation for intervention. *AIDS Behav* 2008; **12** (suppl)**:** S17–24.

a165 Centers for Disease Control and Prevention. AtlasPlus: HIV, hepatitis, STD, TB, social determinants of health data. Data on HIV and primary and secondary syphilis, ages 13–24, 2010. https://www.cdc.gov/nchhstp/atlas/index.htm (accessed Sept 10, 2019).

a166 Ataie M, Nokhodian Z, Ataei B, Kassaian N, Yaran M, Hassannejad R. Seroprevalence of hepatitis B virus and human immunodeficiency virus among young prisoners. *J Res Med Sci* 2013; **18:** 70–72.

a167 Hennessey KA, Kim AA, Griffin V, Collins NT, Weinbaum CM, Sabin K. Prevalence of infection with hepatitis B and C viruses and co-infection with HIV in three jails: a case for viral hepatitis prevention in jails in the United States. *J Urban Health* 2009; **86:** 93–105.

a168 Popov G, Plochev K, Chervenyakova T. 1391 PREVALENCE OF HEPATITIS C VIRUS INFECTION AMONG JUVENILE PRISONERS. *J Hepatol* 2012; **56:** S546.

a169 Thomas AR, Keene WE, Cieslak PR. Seroprevalence of hepatitis B and C in juvenile detention entrants, Oregon, 1994–1996. *J Adolesc Health* 2005; **37:** 410–13.

a170 Wasley A, Kruszon-Moran D, Kuhnert W, et al. The prevalence of hepatitis B virus infection in the United States in the era of vaccination. *J Infect Dis* 2010; **202:** 192–201.

a171 Bair RMB, Baillargeon JG, Kelly PJ, et al. Prevalence and risk factors for hepatitis C virus infection among adolescents in detention. *Arch Pediatr Adolesc Med* 2005; **159:** 1015–18.

a172 Cocoros N, Nettle E, Church D, et al. Screening for Hepatitis C as a Prevention Enhancement (SHAPE) for HIV: an integration pilot initiative in a Massachusetts County correctional facility. *Public Health Rep* 2014; **129** (suppl 1)**:** 5–11.

a173 Feldman GMS, Sorvillo F, Cole B, Lawrence WA, Mares R. Seroprevalence of hepatitis C among a juvenile detention population. *J Adolesc Health* 2004; **35:** 505–08.

a174 Murray KF, Richardson LP, Morishima C, Owens JW, Gretch DR. Prevalence of hepatitis C virus infection and risk factors in an incarcerated juvenile population: a pilot study. *Pediatrics* 2003; **111:** 153–57.

a175 Nokhodian Z, Ataei B, Kassaian N, Yaran M, Hassannejad R, Adibi P. Seroprevalence and risk factors of hepatitis C virus among juveniles in correctional center in Isfahan, Iran. *Int J Prev Med* 2012; **3** (suppl 1)**:** S113–17.

a176 Ogilvie EL, Veit F, Crofts N, Thompson SC. Hepatitis infection among adolescents resident in Melbourne Juvenile Justice Centre: risk factors and challenges. *J Adolesc Health* 1999; **25:** 46–51.

a177 Armstrong GL, Wasley A, Simard EP, McQuillan GM, Kuhnert WL, Alter MJ. The prevalence of hepatitis C virus infection in the United States, 1999 through 2002. *Ann Intern Med* 2006; **144:** 705–14.

a178 Begier EM, Bennani Y, Forgione L, et al. Undiagnosed HIV infection among New York City jail entrants, 2006: results of a blinded serosurvey. *J Acquir Immune Defic Syndr* 2010; **54:** 93–101.

a179 Boisson EV, Trotman C. HIV seroprevalence among male prison inmates in the six countries of the Organization of Eastern Caribbean states in the Caribbean (OECS). *West Indian Med J* 2009; **58:** 106–11.

a180 Rothon DA, Strathdee SA, Cook D, Cornelisse PG. Determinants of HIV-related high risk behaviours among young offenders: a window of opportunity. *Can J Public Health* 1997; **88:** 14–17.

a181 Safdar S, Mehmood A, Abbas SQ. Prevalence of HIV/AIDS among jail inmates in Sindh. *J Pak Med Assoc* 2009; **59:** 111–12.

a182 Sweeney P, Lindegren ML, Buehler JW, Onorato IM, Janssen RS. Teenagers at risk of human immunodeficiency virus type 1 infection. Results from seroprevalence surveys in the United States. *Arch Pediatr Adolesc Med* 1995; **149:** 521–28.

a183 VanHandel M, Beltrami JF, MacGowan RJ, Borkowf CB, Margolis AD. Newly identified HIV infections in correctional facilities, United States, 2007. *Am J Public Health* 2012; **102** (suppl 2)**:** S201–04.

a184 UN Office on Drugs and Crime. HIV prevalence and related risk factors in prison settings in mainland Tanzania: findings from a rapid situational assessment. Vienna, Austria: UN Office on Drugs and Crime, 2013. https://www.unodc.org/wdr2016/ field/7.2_HIV_and_hepatitis_among_people_held_in_prisons_ WDR2016.xls (accessed Sept 10, 2019).

a185 Kim AAM, McFarland W, Kellogg T, Katz MH. Sentinel surveillance for HIV infection and risk behavior among adolescents entering juvenile detention in San Francisco: 1990–1995. *AIDS* 1999; **13:** 1597–98.

a186 Crosby R, Salazar LF, Diclemente RJ, Yarber WL, Caliendo AM, Staples-Horne M. Health risk factors among detained adolescent females. *Am J Prev Med* 2004; **27:** 404–10.

a187 Johnston EE, Argueza BR, Graham C, Bruce JS, Chamberlain LJ, Anoshiravani A. In their own voices: the reproductive health care experiences of detained adolescent girls. *Womens Health Issues* 2016; **26:** 48–54.

a188 Kelly PJ, Lesser J, Paper B. Detained adolescents' attitudes about pregnancy and parenthood. *J Pediatr Health Care* 2008; **22:** 240–45.

a189 Schmiege SJ, Broaddus MR, Levin M, Bryan AD. Randomized trial of group interventions to reduce HIV/STD risk and change theoretical mediators among detained adolescents. *J Consult Clin Psychol* 2009; **77:** 38–50.

a190 Rissel C, Heywood W, de Visser RO, et al. First vaginal intercourse and oral sex among a representative sample of Australian adults: the Second Australian Study of Health and Relationships. *Sex Health* 2014; **11:** 406–15.

a191 Liu G, Hariri S, Bradley H, Gottlieb SL, Leichliter JS, Markowitz LE. Trends and patterns of sexual behaviors among adolescents and adults aged 14 to 59 years, United States. *Sex Transm Dis* 2015; **42:** 20–26.

a192 Voisin DR, Salazar LF, Crosby R, DiClemente RJ, Yarber WL, Staples-Horne M. HIV testing among detained youth. *J HIV AIDS Prev Child Youth* 2006; **6:** 83–96.

a193 Williams RA, Hollis HM. Health beliefs and reported symptoms among a sample of incarcerated adolescent females. *J Adolesc Health* 1999; **24:** 21–27.

a194 Narring F, Michaud PA, Sharma V. Demographic and behavioral factors associated with adolescent pregnancy in Switzerland. *Fam Plann Perspect* 1996; **28:** 232–36.

a195 Yeung A, Aggleton P, Richters J, et al. Sex education: findings from the Second Australian Study of Health and Relationships. *Sex Health* 2017; **14:** 293–95.

a196 Feinstein RA, Lampkin A, Lorish CD, Klerman LV, Maisiak R, Oh MK. Medical status of adolescents at time of admission to a juvenile detention center. *J Adolesc Health* 1998; **22:** 190–96.

a197 Sattler AL. Treating Youths in the Juvenile Justice System. *Pediatr Clin North Am* 2017; **64:** 451–62.

a198 Sedgh G, Finer LB, Bankole A, Eilers MA, Singh S. Adolescent pregnancy, birth, and abortion rates across countries: levels and recent trends. *J Adolesc Health* 2015; **56:** 223–30.

a199 Peres CA, Peres RA, da Silveira F, Paiva V, Hudes ES, Hearst N. Developing an AIDS prevention intervention for incarcerated male adolescents in Brazil. *AIDS Educ Prev* 2002; **14** (5 suppl B)**:** 36–44.

a200 Abma JC, Martinez GM. Sexual activity and contraceptive use among teenagers in the United States, 2011–2015. *Natl Health Stat Report* 2017; **104:** 1–23.

a201 Kann L, McManus T, Harris WA, et al. Youth risk behavior surveillance - United States, 2017. *MMWR Surveill Summ* 2018; **67:** 1–114.

a202 Kreisel K, Torrone E, Bernstein K, Hong J, Gorwitz R. Prevalence of pelvic inflammatory disease in sexually experienced women of reproductive age - United States, 2013–2014. *MMWR Morb Mortal Wkly Rep* 2017; **66:** 80–83.
